# Coagulation factors VII, IX and X are effective antibacterial proteins against drug-resistant Gram-negative bacteria

**DOI:** 10.1038/s41422-019-0202-3

**Published:** 2019-08-09

**Authors:** Jinwu Chen, Xiaojie Li, Ling Li, Ting Zhang, Qing Zhang, Fangming Wu, Diyue Wang, Hongze Hu, Changlin Tian, Dongsheng Liao, Liang Zhao, Danxia Song, Yongyun Zhao, Chuanfang Wu, Xu Song

**Affiliations:** 1 Center for Functional Genomics and Bioinformatics, Key Laboratory of Bio-Resource and Eco-Environment of Ministry of Education, College of Life Sciences, Sichuan University, Chengdu, 610064 Sichuan China; 2 State Key Laboratory of Biotherapy, West China Hospital, Sichuan University, Chengdu, 610041 Sichuan China; 3 High Magnetic Field Laboratory, Chinese Academy of Sciences, Hefei, 230031 Anhui China; 4 CAS key Laboratory of Soft Matter Chemistry, Department of Chemistry, University of Science and Technology of China, Hefei, 230026 Anhui China; 5 Hefei National Laboratory for Physical Sciences at the Microscale and School of Life Sciences, University of Science and Technology of China, Hefei, 230026 Anhui China

**Keywords:** Immunology, Mechanisms of disease

## Abstract

Infections caused by drug-resistant “superbugs” pose an urgent public health threat due to the lack of effective drugs; however, certain mammalian proteins with intrinsic antibacterial activity might be underappreciated. Here, we reveal an antibacterial property against Gram-negative bacteria for factors VII, IX and X, three proteins with well-established roles in initiation of the coagulation cascade. These factors exert antibacterial function via their light chains (LCs). Unlike many antibacterial agents that target cell metabolism or the cytoplasmic membrane, the LCs act by hydrolyzing the major components of bacterial outer membrane, lipopolysaccharides, which are crucial for the survival of Gram-negative bacteria. The LC of factor VII exhibits in vitro efficacy towards all Gram-negative bacteria tested, including extensively drug-resistant (XDR) pathogens, at nanomolar concentrations. It is also highly effective in combating XDR *Pseudomonas aeruginosa* and *Acinetobacter baumannii* infections in vivo. Through decoding a unique mechanism whereby factors VII, IX and X behave as antimicrobial proteins, this study advances our understanding of the coagulation system in host defense, and suggests that these factors may participate in the pathogenesis of coagulation disorder-related diseases such as sepsis via their dual functions in blood coagulation and resistance to infection. Furthermore, this study may offer new strategies for combating Gram-negative “superbugs”.

## Introduction

Bacterial pathogens pose one of the most urgent global health threats as a result of their growing resistance to current antibacterial drugs.^1,2^ Compared to the Gram-positive bacteria, the Gram-negative bacteria are harder to kill because of the presence of the outer membrane, which contributes to the reduced cell permeability and serves as a mechanism for the antibiotic resistance.^3,4^ Bacterial infections, especially those caused by the Gram-negative pathogens, have aroused wide concern due to the lack of effective antimicrobial reagents.^4–6^ Recently, the World Health Organization (WHO) listed 12 bacteria that pose the greatest threat to human health because of their resistance to antibiotics; among them, three carbapenem-resistant Gram-negative bacteria, *Acinetobacter baumannii*, *Pseudomonas aeruginosa* and *Enterobacteriaceae*, are considered to be of critical priority.^7^ Many efforts have been made to address this healthcare issue. However, certain natural mammalian proteins that possess an intrinsic antibacterial property might be underappreciated.

Factor VII (FVII) is a single-chain serine protease zymogen that circulates in blood and can bind the receptor tissue factor (TF) with high affinity and specificity. When bleeding occurs, TF in the blood vessel wall is exposed to FVII. Once bound to TF, FVII is activated into FVIIa, forming a TF-FVIIa complex that activates factor IX (FIX) and factor X (FX), leading to thrombin generation and ultimately clotting.^8^ Following the activation process, the coagulation factors are cleaved to form two chains, including a light chain (LC) and a heavy chain (HC), which are connected to each other by a single disulfide bond. The HC possesses serine protease activity, whereas the LC consists of a γ-carboxyglutamic acid domain (Gla) and two epidermal growth factor-like domains (EGF1 and EGF2), responsible for the target recognition.^9,10^

Our previous studies have demonstrated that overexpression of TF in tumor cells is related to high-level metastasis,^11^ and that the LC of FVII (lFVII) can be designed to serve as a molecular vehicle for the development of a targeted therapeutic agent based on its interaction with TF.^12^ Recently, we unexpectedly discovered a direct antibacterial activity towards Gram-negative *Escherichia coli* for lFVII; this strongly suggests that FVII is multifunctional and not merely an initiator of blood coagulation. Following on from the initial findings, we set out to determine the molecular target and specific mechanism through which lFVII kills bacteria, and found that lFVII acts by destroying the bacterial cell envelope and hydrolyzing the major components of bacterial outer membrane, lipopolysaccharides (LPS). To further define the antibacterial activity exhibited by lFVII, we assessed the in vitro activity of lFVII towards a range of Gram-negative bacteria other than *E. coli*, including the extensively drug-resistant (XDR) clinical isolates, and evaluated its effectiveness in combatting XDR Gram-negative bacterial infections in vivo. Our data show that lFVII is highly effective against all bacteria tested, consistent with the notion that LPS is crucial for the survival of Gram-negative bacteria.^13,14^ In addition, we tried to extend our finding to FIX and FX, two other coagulation factors with well-established roles in initiation of clotting.

## Results

### FVII, FIX, FX and their LCs exert antibacterial effects

**Fig. 1:**
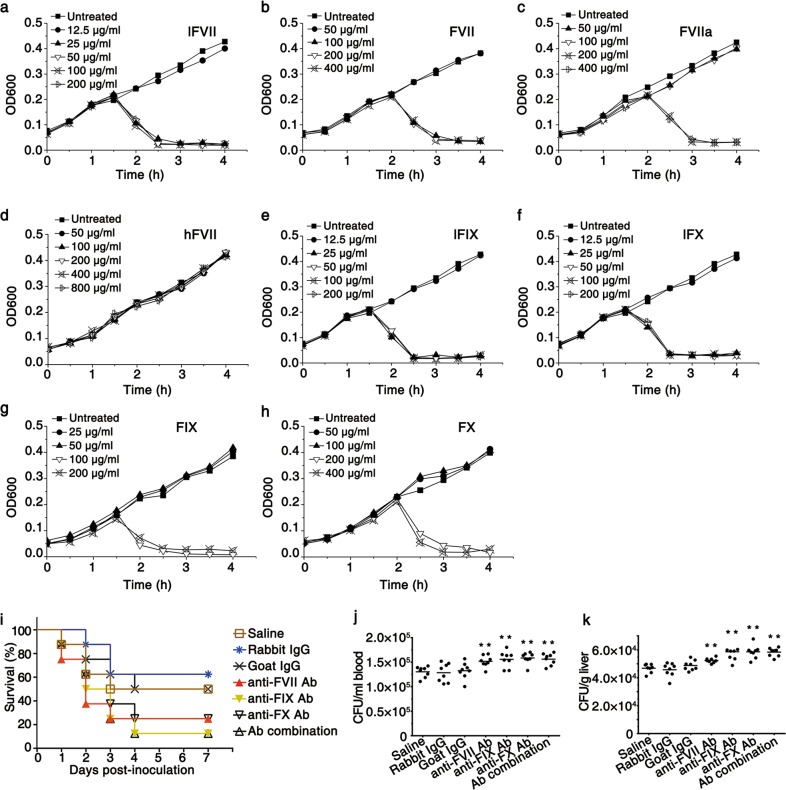
FVII, FIX, FX, and their LCs exhibit antibacterial activity. **a**–**h** Growth kinetic measurements of *E. coli* DH5α exposed to different concentrations of lFVII (**a**), FVII (**b**), FVIIa (**c**), hFVII (**d**), lFIX (**e**), lFX (**f**), FIX (**g**) and FX (**h**). Untreated bacteria were included in all experiments. Data represent mean values from five or more independent experiments. **i** Resistance to *P. aeruginosa* in BALB/c mice was weakened by neutralizing endogenous coagulation factors. The following antibodies (dose per mouse in parentheses), with saline as a blank control, were investigated in groups of eight mice: anti-mFVII Ab (1 µg), anti-mFIX Ab (8 µg), anti-mFX Ab (8 µg), Ab combination (1 µg of anti-mFVII Ab, 8 µg of anti-mFIX Ab and 8 µg of anti-mFX Ab), normal goat IgG (16 µg) and normal rabbit IgG (16 µg). After 1 h of antibody injections, each mouse was intravenously inoculated with 2 × 10^5^ CFU of *P. aeruginosa* L93. **j**, **k** Increased bacterial load in blood (**j**) and liver (**k**) caused by neutralizing endogenous coagulation factors. Mice were treated as described in **i**. Bacterial titers remaining in blood and liver were assayed at 2 h post inoculation of *P. aeruginosa*. Bars indicate the means (*n* = 8). **P < 0.01, significantly different from the saline-injected group

In this study, the LCs of FVII, FIX and FX were produced by *E. coli* BL21 (DE3), and their intact forms, as well as the HC of FVII, were produced by CHO-DG44 cells. Antibacterial effects of these proteins were first evaluated by determining the minimum inhibitory concentrations (MICs) towards *E. coli* DH5α. Growth kinetic measurements showed that lFVII, which had a MIC of 25 µg/mL, as well as intact FVII and FVIIa (activated in vitro^15^), strongly inhibited the growth of bacteria, whereas the HC of FVII (hFVII) did not exhibit any inhibitory effect (Fig. 1a–d). The LCs of FVII, FIX and FX, which share an analogous domain structure,^9,10^ are conserved at the amino acid level (Supplementary information, Fig. S1a); equivalent levels of antibacterial activity were identified in the LC of FIX (lFIX), the LC of FX (lFX), as well as the intact FIX and FX (Fig. 1e–h). These results indicated that the LCs derived from the coagulation factors are critical for the antibacterial activity. In addition, our results showed that the antibacterial activity of FVII, FIX, and FX can be blocked by specific antibody (Supplementary information, Fig. S1b), ruling out potential contamination by certain proteins with antibacterial activity during purification process.

After identifying the antibacterial activity of the recombinant LCs towards Gram-negative *E. coli* DH5α in vitro, we carried out a loss-of-function assay to assess involvements of the endogenous coagulation factors in animal’s resistance to *Pseudomonas aeruginosa*, which is a leading Gram-negative opportunistic pathogen in most public healthcare settings.^16^ Using the normal IgGs and saline as controls, antibodies specific for the coagulation factors were injected intravenously to neutralize the endogenous coagulation factors in BALB/c mice. The injected mice were then intravenously inoculated with *P. aeruginosa* L93, a clinical isolate obtained from a hospital in Sichuan, China. The coagulation factor-neutralized mice showed a reduced survival rate (Fig. 1i). Furthermore, a visible increase in the number of viable *P. aeruginosa* L93, which was determined by growth on selective media,^17^ was observed in blood and liver tissues of coagulation factor-neutralized mice compared to the mice in control groups (Fig. 1j, k). The mice infused with FVII- and FIX-specific antibodies were shown to have no change in the prothrombin time (PT) and activated partial thromboplastin time (aPTT), and the anti-FX Ab-infused mice exhibited a slight increase in aPTT (Supplementary information, Fig. S1c). These results indicate that the infused antibodies did not influence animals’ coagulation status. Therefore, our results are consistent with, and possibly account for, the observations that the bacterial infectious diseases sepsis and pneumonia are, respectively, the first (~38% of cases) and fourth (~25% of cases) leading causes of death in patients with hemophilia B, one of the main types of hemophilia caused by FIX deficiency,^18^ and that isolated acquired FVII deficiency appears to be associated with several morbid conditions such as severe systemic sepsis.^19,20^ Collectively, our results, along with the previous clinical data, hint an intrinsic antibacterial property for the endogenous coagulation factors in organism.

### LCs damage bacterial cell envelope

Based on the finding that lFVII is more stable than intact FVII (data not shown), we used the LCs to investigate the antibacterial mechanism of these coagulation factors. Growth kinetic measurements showed that the LCs (at four times their MIC) exhibited kinetic behaviors similar to those of cell envelope-interfering agents (such as penicillin and ampicillin) but different from membrane-active agents (such as polymyxin B and novispirin G10) that cause rapid cell lysis (Supplementary information, Fig. S2a).^21^ Killing kinetics also showed that bacterial cells exposed to the LCs underwent a slow multiplication at the beginning of the culturing process (0–1.5 h), followed by a rapid decline (Supplementary information, Fig. S2b). These results indicated that the LCs possess a similar antibacterial pattern to cell envelope-interfering agents, and might act by targeting the bacterial cell envelope.

**Fig. 2:**
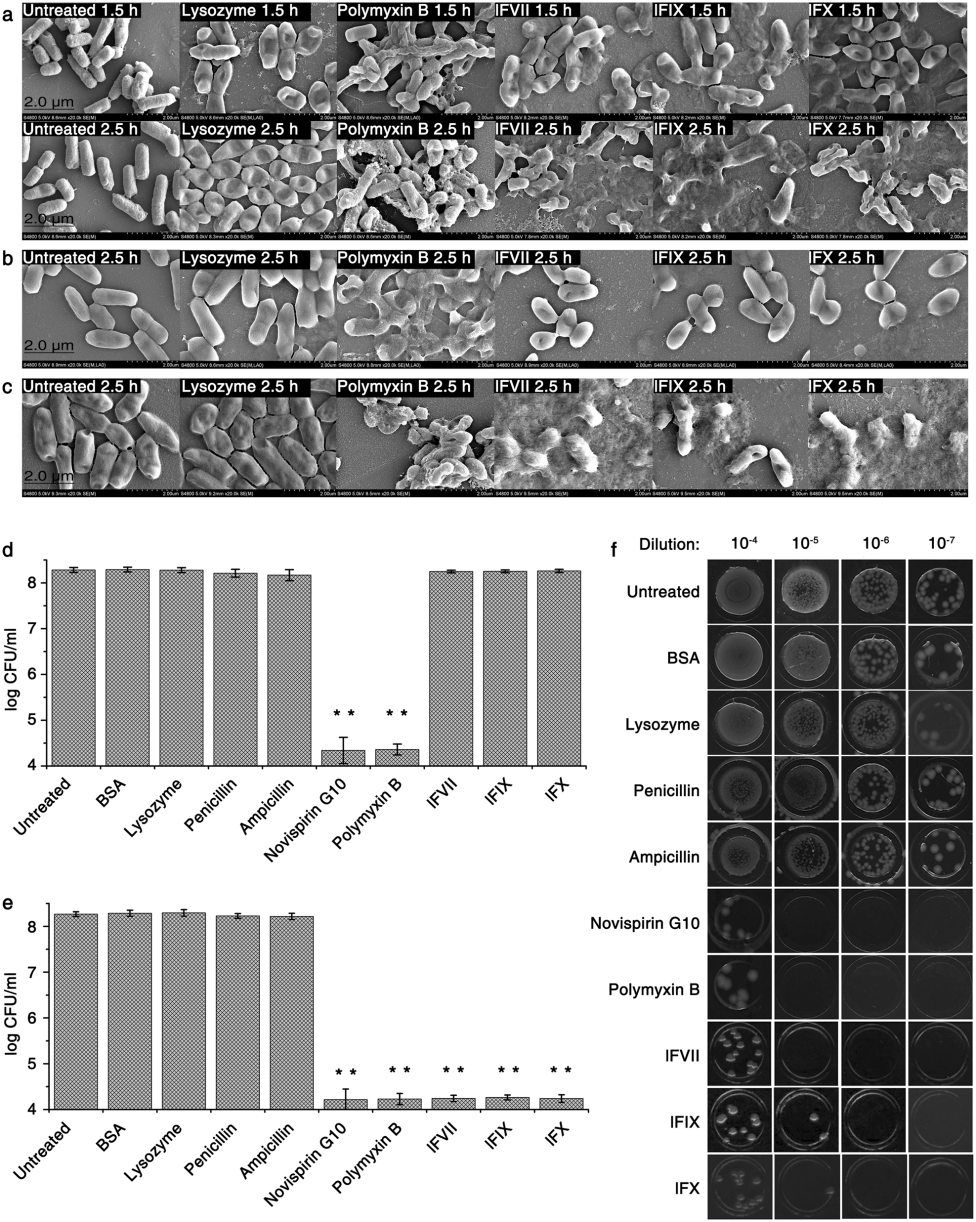
LCs damage bacterial cell envelope without disturbing its biosynthesis. **a**–**c** SEM images of *E. coli* DH5α following treatment with different agents under different culture conditions. Bacteria were incubated with the indicated agents in LB for 1.5 or 2.5 h (**a**), in hypertonic LB for 2.5 h (**b**) or in hypotonic LB for 2.5 h (**c**), and then the pictures were captured using SEM. **d**–**f** Effects of the LCs on the survival of starving *E. coli* DH5α. Bacteria were treated with the indicated agents in hypertonic (**d**) or hypotonic TBS (**e**) for 4 h at 37 °C. Error bars represent SD (*n* = 3). **P < 0.01, significantly different from the untreated cells. Bacteria treated in hypotonic TBS, which were the same as those tested in **e**, were also spotted on hypotonic LB solid medium following a serial dilution (**f**). Untreated bacteria were included in all experiments. The final concentrations of different agents are listed as follows: lysozyme, 50 μg/mL; polymyxin B, 10 μg/mL; lFVII, 100 μg/mL (4× MIC against *E. coli* DH5α); lFIX, 100 μg/mL (4× MIC against *E. coli* DH5α); lFX, 100 μg/mL (4× MIC against *E. coli* DH5α); BSA, 100 μg/mL; penicillin, 100 μg/mL; ampicillin, 250 μg/mL; novispirin G10, 10 μg/mL

To further determine whether the bacterial death caused by the LCs was related to cell envelope damage, the morphological features of *E. coli* DH5α cells in lysogeny broth (LB) were investigated both in the presence and absence of the LCs by scanning electron microscopy (SEM). The results showed that a 1.5-h treatment with the LCs led to an apparent irregularity and collapse in cell shape, and an extended treatment for 2.5 h caused more serious cell damage, as indicated by the appearance of abundant membrane material and the inner contents of the bacteria (Fig. 2a). Treatment for 4 h resulted in complete cell damage, and the cell debris could barely be observed (data not shown). In contrast, some of the polymyxin B-treated *E. coli* cells retained their shape in LB because of support from the cell envelope, even though the bacterial cytoplasmic membrane had been destroyed (Fig. 2a).^22^ In addition, a minor morphological change was observed in the lysozyme-treated cells (Fig. 2a). Although lysozyme can target and degrade the peptidoglycan (PGN) in cell envelope, it was reported to exhibit only a weak damaging effect on the cell envelope of Gram-negative bacteria.^23^

The LC-caused cell envelope damage was also analyzed in hypertonic and hypotonic LB. Different from the LC treatment in isotonic LB that caused complete cell lysis (Fig. 2a), the same treatment in hypertonic LB caused only a minor change in cell shape, with cells becoming slightly more spherical (Fig. 2b). Given that the bacterial cytoplasmic membrane remained intact in hypertonic broth when the cell envelope was destroyed,^24^ the results further suggest that the LC treatment causes the cell envelope to collapse but does not influence the integrity of the cytoplasmic membrane, which remains intact. This was also supported by the results obtained from the same assay in the hypotonic LB, where the LC-treated cells suffered significant damage (Fig. 2c). Following the destruction of the bacterial cell envelope in the hypotonic broth, the intracellular osmotic pressure would cause the cells to burst,^24^ giving results similar to those observed in the isotonic broth (Fig. 2a).

### Efficacy of the LCs against nutrient-starved bacteria

Most antibiotics are inert in killing Gram-negative bacteria under growth-compromising conditions such as nutrient starvation.^25^ Here, we proceeded to address whether the cell damage caused by LCs occurs in starving bacteria. The testing system involved immersing *E. coli* DH5α cells in Tris-buffered saline (TBS) containing different agents, including the LCs, and the level of cell damage was measured by colony formation assay.

The test was first carried out in hypertonic TBS, where the starving *E. coli* DH5α cells exhibited resistance to the cell envelope-interfering agents penicillin and ampicillin as expected (Fig. 2d).^25^ Treatment with membrane-active agents, including polymyxin B and novispirin G10, significantly reduced colony-forming unit (CFU) counts because of the membrane disruption-induced release of the inner contents of the bacterial cells (Fig. 2d).^26^ The LC treatments, however, were barely able to affect the CFU counts (Fig. 2d), which seemed consistent with those obtained from the hypertonic LB (Fig. 2b).

A similar experiment involving the use of hypotonic TBS instead of hypertonic TBS was conducted to determine whether the LC-treated cells would show similar levels of damage to those in the hypotonic LB. In this condition, the cell envelope-interfering agents penicillin and ampicillin were still functionally inert; the LC treatment, however, significantly repressed the bacterial survival (Fig. 2e, f). The efficacy of the LCs against starving bacteria suggested that the bacterial metabolism, especially the cell envelope biosynthesis, which is paused under starvation conditions, could not be the target of the LCs, and that the LCs might function as cell envelope-active agents, damaging the bacterial cell envelope in a metabolism-independent manner.

On the other hand, the data presented here (Fig. 2d–f), together with the SEM images shown in Fig. 2a–c, demonstrated that the cell envelope-active agent lysozyme can hardly influence bacterial viability both under starvation stresses and under favorable growth conditions. This raises an interesting possibility that the LCs may target certain components of the bacterial cell envelope other than PGN, which is a well-characterized lysozyme target.^27^

### lFVII hydrolyzes LPS and lipid A

To further confirm that PGN is not the LC target, we incubated the LCs, using lysozyme as a positive control, with the purified PGN from different bacterial strains (Invivogen). SEM detection of the treated PGN showed that PGN was degraded by lysozyme, and the LC treatment did not cause any degradation of the tested PGN (Supplementary information, Fig. S3).

We proceeded to determine the LC-specific target, and then focused on the bacterial outer membrane, which is an important phospholipid- and polysaccharide-containing component of Gram-negative cell envelope lying outside the PGN layer.^3^ LPS are major components of bacterial outer membrane and are crucial for the survival of Gram-negative bacteria.^13,14^ With this in mind, we turned our attention to LPS to investigate its involvement in the bacterial damage caused by LCs. The possibility of an interaction between the LCs and the LPS from *E. coli* K12 (Invivogen) was examined using a dansyl-polymyxin (DPX) binding assay.^28^ It was possible to observe binding of the LCs to the LPS because the binding of DPX to LPS was attenuated by addition of the LCs (Supplementary information, Fig. S4a, middle panel). In addition, the electrophoretic profiles examined by silver staining showed that overnight treatment with any of the LCs at 37 °C led to the degradation of *E. coli* K12 LPS (Supplementary information, Fig. S4b).

**Fig. 3:**
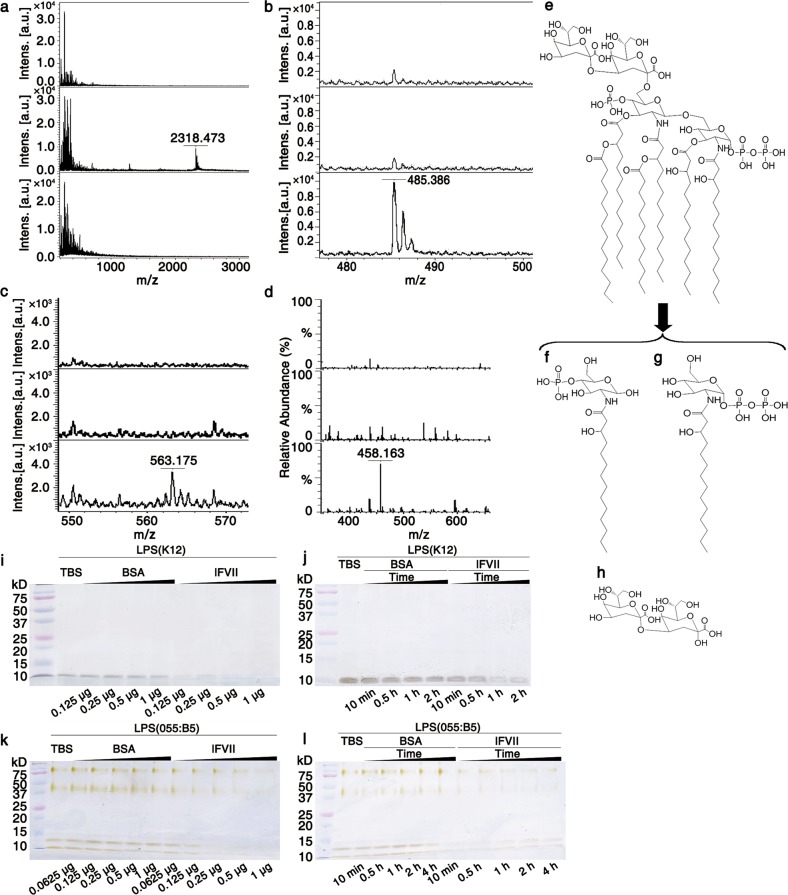
lFVII-triggered LPS hydrolysis. **a**–**d** lFVII-triggered *E. coli* K12 LPS hydrolysis detected by MALD/TOF-MS (**a**–**c**) and ESI-MS (**d**). lFVII in buffer (150 µg/mL) (top); LPS in buffer (625 µg/mL) (middle); LPS (625 µg/mL) incubated with lFVII (150 µg/mL) in buffer (bottom). **e**–**h** Structures of 6-Kdo-(1-bis-4′-mono)-phosphorylated-hexaacylated-lipid A (the peak with an *m/z* value of 2318.473 in **a**, **e**) and the LPS hydrolysates produced after lFVII treatment (**f**–**h**). **i**–**l** lFVII degrades the LPS from *E. coli* K12 (**i**, **j**) and *E. coli* 055:B5 (**k**, **l**). Twenty micrograms of *E. coli* K12 LPS or 10 μg of *E. coli* 055:B5 was tested at 37 °C per reaction; the dose of protein (**i**, **k**) and the treatment time (**j**, **l**) were increased as indicated; the treatment time in **i** and **k** was 2 h, and the protein doses in **j** and **l** were 0.25 μg and 1 μg per reaction, respectively. Separated LPS samples on Tricine-SDS-PAGE were examined by silver staining

Following on from the initial experiments, we took lFVII as an example to further determine whether the LC treatment triggered LPS hydrolysis by detecting changes in the spectrum of LPS using matrix-assisted laser desorption/ionization time of flight mass spectrometry (MALDI/TOF-MS). The spectrum of the *E. coli* K12 LPS showed an isotopic peak with an *m/z* value of 2318.473 (Fig. 3a, middle panel), corresponding to 6-Kdo-(1-bis-4′-mono)-phosphorylated-hexaacylated-lipid A (Fig. 3e).^29^ This peak was no longer observed following the lFVII treatment (Fig. 3a, bottom panel). Instead, the lFVII-treated LPS produced two different peaks with *m/z* values of 485.386 and 563.175 (Fig. 3b, c, bottom panels), which could be attributed to the glucosamine backbone structures that had been either monophosphorylated at their O-4 position or bisphosphorylated at their O-1 position and substituted with 14:0 (3-OH) at the N-2 position (Fig. 3f, g). The lFVII-treated *E. coli* K12 LPS was also detected by electrospray ionization mass spectrometry (ESI-MS) in positive ion mode, and the peak obtained at *m/z* 458.163 (Fig. 3d, bottom panel) could be attributed to Kdo2 (Fig. 3h). Michaelian saturation kinetics were observed for lFVII towards *E. coli* K12 LPS under the optimum conditions of 37 °C and pH 7.4 (Supplementary information, Fig. S4c–f), and the *K*_*m*_ and *V*_max_ values for lFVII with *E. coli* K12 LPS were determined as 0.165 ± 0.009 mM and 0.063 ± 0.004 µmol/min/mg, respectively. In addition, lFVII was revealed to hydrolyze *E. coli* K12 LPS in a dose- and time-dependent manner (Fig. 3i, j). Since *E. coli* K12 LPS is O-antigen deficient, we next examined whether lFVII can hydrolyze the *E. coli* 055:B5 LPS (Sigma-Aldrich), which has O-antigen. A dose- and time-dependent manner was also revealed for the lFVII-mediated hydrolysis of the O-antigen-containing LPS (Fig. 3k, l). Taken together, these results indicated that lFVII could be an LPS hydrolase.

**Fig. 4:**
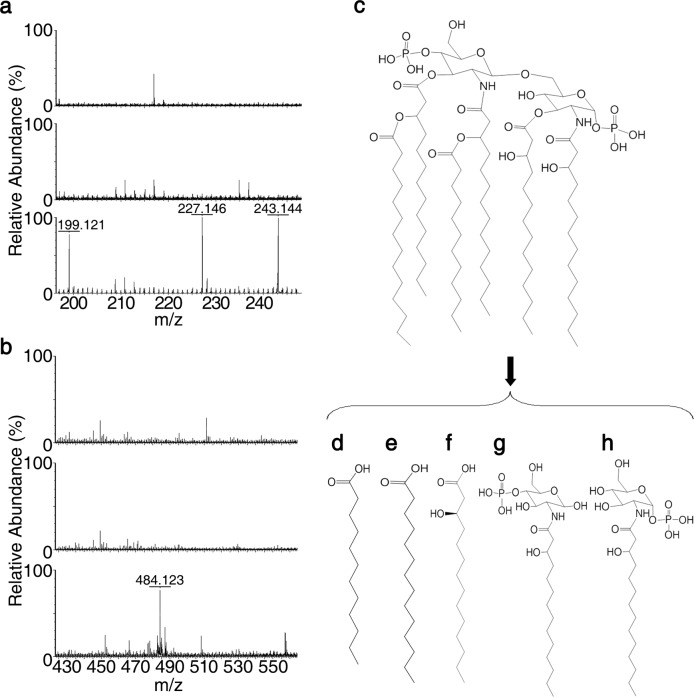
lFVII-triggered hydrolysis of *E. coli* F583 lipid A. **a**, **b** lFVII-triggered lipid A hydrolysis detected by ESI-MS. lFVII in buffer (150 µg/mL) (top); lipid A in buffer (700 µg/mL) (middle); lipid A (700 µg/mL) incubated with lFVII (150 µg/mL) in buffer (bottom). **c**–**h** Structures of *E. coli* F583 lipid A (**c**) and the lipid A hydrolysates produced after lFVII treatment (**d**–**h**)

The minimum requirement of LPS for growth of *E. coli* includes the Kdo and lipid A domains, and the latter is responsible for the toxic effects associated with Gram-negative bacterial infection.^30,31^ We had detected the potential Kdo2 and phosphorylated acylated glucosamine backbone structures from the lFVII-produced LPS hydrolysates (Fig. 3). Therefore, lipid A was employed to further detect the potential lFVII-targeted hydrolysis sites. Lipid A of *E. coli* F583 (*m/z* 1796.224, Sigma-Aldrich) was treated with lFVII and subsequently analyzed by ESI-MS in negative ion mode. Four molecular ion peaks were observed with *m/z* values of 199.121, 227.146, 243.144 and 484.123, respectively (Fig. 4a, b, bottom panels). These ions were attributed to the 12:0 fatty acid (*m/z* 199.121) (Fig. 4d), 14:0 fatty acid (*m/z* 227.146) (Fig. 4e), R-14:0 (3-OH) fatty acid (*m/z* 243.144) (Fig. 4f) and 1- or 4-monophosphorylated-2-N-β-(R)-hydroxyl-myristoyl-GlcN (*m/z* 484.123) (Fig. 4g, h), which resulted from the hydrolysis of 12:0 (3-(R)-O-12:0) at the N-2′ position, 14:0 (3-O-14:0) at the O-3′ position, 14:0 (3-OH) at the O-3 or O-3′ position, and β-D-GlcN4P-(1′→6)-α-D-GlcN1P at the O-1′ or O-6 position, respectively.

Collectively, the above data demonstrated that lFVII attacks Gram-negative bacteria by hydrolyzing LPS in the bacterial cell envelope. We hypothesize that the same principle could also be applicable to lFIX and lFX, because these three LCs are conserved at the amino acid level and share an analogous domain structure (Supplementary information, Fig. S1a).^9,10^

### Residues critical for the antibacterial activity of lFVII

**Fig. 5:**
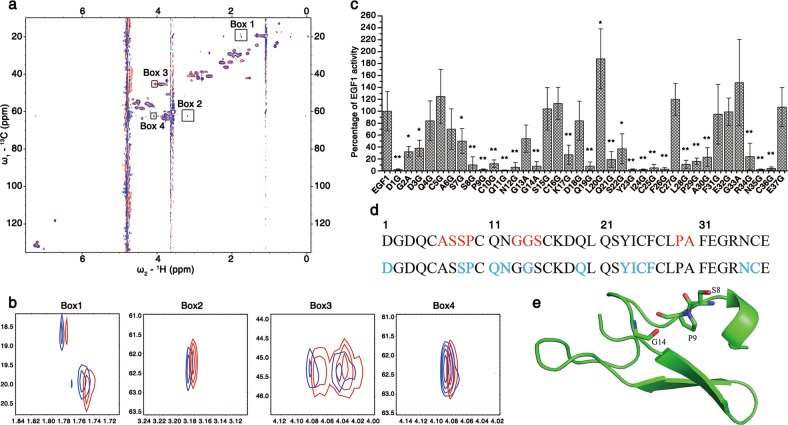
Residues critical for the antibacterial activity of lFVII. **a**, **b** lFVII EGF1-LPS interaction detected by NMR chemical shift perturbation experiments. Two-dimensional ^1^H–^13^C HSQC spectra were recorded in the presence of different molar ratios of EGF1 to LPS (Red, 1:0, Blue, 1:0.1) (**a**). When the molar ratio between EGF1 and LPS reached 1:0.25, all the signals disappeared (data not shown), possibly due to molecular aggregation. Four different regions of the spectra named Boxes 1–4 were enlarged in **b**. The peaks in Box 1 showed a ^13^C chemical shift value (CSV) of ~20 ppm, which clearly indicates that the signal comes from the Cβ nucleus of an alanine; the peak in Box 2 showed a ^13^C CSV of ~62 ppm, and a ^1^H CSV of 3.2 ppm, which could possibly arise from Cβ–Hβ of a serine or threonine (since there is no threonine in the primary sequence of EGF1, it has to be serine); the peaks in Box 3 showed a ^13^C CSV of ~20 ppm, which directly points to a glycine residue; the peak in Box 4 showed a ^13^C CSV of ~62 ppm, and a ^1^H CSV of 4.1 ppm, which could possibly arise from Cα–Hα of a proline or valine (it could be proline as there is no valine in the EGF1 sequence). **c** Antibacterial activities of a serial EGF1 mutants towards *E. coli* DH5α. To produce EGF1 mutants, alanine was introduced to replace the glycine in EGF1 sequence, and glycine was introduced to replace the amino acids other than glycine. The antibacterial activities were determined by MIC measurement, and the data were presented as a percentage of the activity of wild-type EGF1 (EGF1) and as means ± SD (*n* = 5 independently purified protein samples). *P < 0.05 and **P < 0.01, significantly different from the EGF1. The value of <10% was considered as a significant reduction in antibacterial activity. **d** Primary sequence of EGF1 domain of FVII. Residues that were perturbed in the NMR titration assay are shown in red, and the residues exhibiting functional importance in antibacterial assay are colored in blue. **e** Crystal structure of the EGF1 domain of FVII (PDB ID: 1QFK).^50^ Side-chains of the potential LPS-interacting residues were displayed. The crystal structure of EGF1 domain used here is generally similar to its solution structure (PDB ID: 1BF9 and 1F7M) (Supplementary information, Fig. S6f)

The above data indicate that lFVII kills *E. coli* by hydrolyzing LPS; moreover, DPX binding assay has detected a potential interaction between lFVII and LPS (Supplementary information, Fig. S4a). Next, proteins of three domains of lFVII, namely Gla, EGF1 and EGF2 (Supplementary information, Fig. S5a), were produced to determine the active region. The antibacterial assays clearly showed that the EGF1 and EGF2 domains were functional, whereas the Gla domain was inactive (Supplementary information, Fig. S5b–d). EGF1 was used for the subsequent investigations. NMR chemical shift perturbation experiments were employed to verify the interaction between EGF1 and LPS. The ^1^H–^13^C HSQC spectra of EGF1 revealed that several peaks had chemical shift changes in the presence of LPS, while most of chemical shifts were not changed (Fig. 5a). These features strongly indicate that LPS can specifically interact with EGF1, as non-specific interaction would cause a wider range of changes.

To find out the active site of EGF1, the chemical shift perturbation data were carefully examined. The shifted peaks were enlarged (Fig. 5b). Based on the primary sequence of EGF1 and the chemical shift values, the peaks in Boxes 1–4 could be, respectively, assigned as alanines (Cβ–Hβ), serine (Cβ–Hβ), glycines (Cα–Hα), and proline (Cα–Hα) (Detailed in Materials and Methods and figure legend). Since it is quite impossible that the interaction interfered with one residue in the sequence, leaving its neighbors unaffected, we examined the sequence of EGF1 for amino acid pairs that are composed of the abovementioned four types of amino acid. These amino acids were shown in red in the first line of Fig. 5d. On the other hand, a serial amino acid mutations were introduced into EGF1 sequence for antibacterial assays (Fig. 5c), and the residues that significantly reduce the antibacterial activity of EGF1 upon mutation were shown in blue in the second line of Fig. 5d. By comparing the colored residues in Fig. 5d, we found that the residues S8, P9 and G14, which are specified in the structured EGF1 domain of FVII (Fig. 5e), appeared to be important in NMR titration as well as in function assay. EGF1 mutant P9G was further selected to test the activity in hydrolyzing LPS. As revealed by the electrophoretic profile, relative to wild-type EGF1, the P9G mutant exhibited a weaker ability to hydrolyze *E. coli* K12 LPS (Supplementary information, Fig. S5e). Therefore, the S8, P9 and G14 sites within EGF1 might be critical for the LPS hydrolysis.

In addition to the residues mentioned above, residues in sequences Q_11_N_12_, Y_23_I_24_C_25_F_26_ and N_35_C_36_ are also important for the antibacterial activity of EGF1 (Fig. 5c). We then carefully examined the peaks of the side-chains of these six amino acid types (2 cysteines and 2 asparagines). NMR titration experiment showed that side-chains of tyrosine and phenylalanine (Supplementary information, Fig. S6a) as well as asparagine and cysteine (Supplementary information, Fig. S6b), and glutamine (Supplementary information, Fig. S6c) were nearly not interfered. On the other hand, side-chains of isoleucine (Supplementary information, Fig. S6d) as well as glycine (Fig. 5b, Box 3) were obviously perturbed in titration. It was possible that mutation of these residues would disrupt the 3D structure of EGF1. First, Y_23_I_24_C_25_F_26_ locate at the second β-strand of EGF1, and mutation of this region would obviously impair the structural stability of the two-stranded β-sheet. Second, mutations of residues Q_11_N_12_ and N_35_C_36_ would disrupt the hydrogen bond network (N35-Q11, N35-Y23, and N12-C36, Supplementary information, Fig. S6e), which would also impair the structural stability.

### In vitro efficacy of lFVII against Gram-negative bacteria

There were two Gram-negative bacterial strains that had been tested for sensitivity to killing at the beginning of this research. After revealing induction of the LPS hydrolysis as the underlying effector mechanism of lFVII, we tried to address whether lFVII possesses a general antibacterial activity against Gram-negative bacteria by determining its minimum bactericidal concentrations (MBCs) towards a range of Gram-negative bacteria, which include both wild-type and clinically isolated bacteria and include both drug-sensitive and drug-resistant bacteria strains.

The assays were initially carried out in Mueller–Hinton broth (MHB),^32^ a nutrient-rich bacterial growth medium. lFVII exhibited potent activity towards all the tested drug-sensitive bacterial strains (MBC < 500 nM), including the wild-type *A. baumannii*, *Klebsiella pneumoniae* and *P. aeruginosa*, as well as the clinically isolated *Enterobacter cloacae* (Table 1). Carbapenem-non-susceptible *A. baumannii* and *P. aeruginosa* are listed by the WHO as the top drug-resistant pathogens that pose the greatest threat to human health.^7^ Noticeably, our data revealed that lFVII was also highly effective against a series of XDR clinical isolates of *A. baumannii* and *P. aeruginosa*,^33^ yielding MBC values ranging from 363 to 874 nM (Table 1). It is noteworthy that lFVII exhibited a similar order-of-magnitude MBC values against all bacteria tested, implying that the antibacterial efficacy of lFVII might be general rather than species specific for Gram-negative bacteria.

**Table 1:** Antibacterial activities of lFVII, polymyxin B and meropenem against a range of Gram-negative bacteria

Bacterial type	Code/name	lFVII (nM)		Polymyxin B (nM)		Meropenem (nM)	
		MHB	TBS	MHB	TBS	MHB	TBS
*A. baumannii*	ATCC 19606	462.49 ± 66.55	10.23 ± 2.22	422.57 + 115.25	1843.94 ± 76.83	453.70 ± 3.43	>450,000
*K. peneumoniae*	ATCC 4352	409.63 ± 34.01	9.84 ± 3.44	514.77 + 184.39	1690.28 ± 153.66	460.56 ± 19.43	>450,000
*P. aeruginosa*	ATCC 27853	411.46 ± 13.93	15.26 ± 4.41	457.14 + 157.50	3726.30 ± 268.91	811.41 ± 102.85	>450,000
*E. cloacae*	Y1	449.01 ± 37.59	12.59 ± 0.17	414.89 + 107.56	1613.45 ± 76.83	342.85 ± 114.28	>450,000
*P. aeruginosa*	PA3 (XDR)	449.01 ± 37.59	12.59 ± 0.17	418.73 ± 119.09	2185.84 ± 733.73	NT	NT
*P. aeruginosa*	PA4 (XDR)	528.27 ± 164.03	12.00 ± 0.19	422.57 ± 107.56	2381.76 ± 845.14	NT	NT
*A. baumannii*	Ab3 (XDR)	373.01 ± 21.87	4.27 ± 1.38	145.98 ± 53.78	1759.43 ± 145.98	NT	NT
*A. baumannii*	Ab5 (XDR)	874.06 ± 16.33	7.67 ± 2.82	276.59 ± 46.10	3518.85 ± 215.13	NT	NT
*A. baumannii*	Ab16 (XDR)	363.38 ± 3.72	3.25 ± 0.33	145.98 ± 46.10	1805.53 ± 268.91	NT	NT
*A. baumannii*	Ab18 (XDR)	375.23 ± 2.62	4.66 ± 1.58	145.98 ± 38.42	1567.35 ± 84.51	NT	NT
*A. baumannii*	Ab30 (XDR)	413.65 ± 41.20	5.13 ± 2.00	138.30 ± 30.73	1982.24 ± 61.46	NT	NT
*A. baumannii*	Ab35 (XDR)	374.03 ± 10.54	5.00 ± 1.91	149.05 ± 4.61	1528.93 ± 69.15	NT	NT
*A. baumannii*	Ab42 (XDR)	401.26 ± 43.51	4.01 ± 0.95	142.14 ± 34.57	1759.43 ± 7.68	NT	NT
*A. baumannii*	Ab46 (XDR)	377.36 ± 4.72	3.49 ± 0.55	130.61 ± 53.78	1882.36 ± 345.74	NT	NT
*A. baumannii*	Ab48 (XDR)	365.30 ± 5.52	3.35 ± 0.32	130.61 ± 46.10	2593.04 ± 1018.01	NT	NT

The MBC value is defined as the minimum drug concentration that causes quantitative bacterial cell death; further details are provided in Materials and Methods. All data are expressed as means ± SD (*n* = 4); NT, not tested. Bacteria with ATCC code were obtained from American Type Culture Collection; the other tested bacteria are clinical isolates obtained from a hospital in Sichuan, China

Since lFVII was found to also repress the nutrient-starved *E. coli* DH5α (Fig. 2e, f), we next repeated the above assays using TBS instead of MHB (Table 1). The comparator polymyxin B had preferential activity towards bacteria in MHB (MBC < 514 nM) over those in TBS (MBC > 1528 nM). However, our data revealed an opposite antibacterial manner for lFVII; that is, lFVII exhibited an order-of-magnitude lower MBC values (<15 nM) against all bacterial strains tested in TBS compared to that in MHB. In contrast, another comparator meropenem, a widely used carbapenem antibiotic,^34^ displayed no activity towards any of the drug-sensitive bacteria in TBS until reaching the concentration of 450 µM.

To further strengthen the antibacterial mechanism proposed for lFVII, we purified LPS from the XDR strains *P. aeruginosa* PA4 and *A. baumannii* Ab3 for LPS hydrolysis analysis. As with the LPS from *E. coli* K12 and *E. coli* 055:B5, the electrophoretic profile revealed that the LPS from XDR strains was also hydrolyzed by lFVII (Supplementary information, Fig. S7). Importantly, our results showed that the LPS-deficient *A. baumannii*, which was isolated from the XDR *A. baumannii* Ab3, exhibited a significant reduction of sensitivity to lFVII (Supplementary information, Table S1). Therefore, our data collectively suggest that, through triggering the hydrolysis of LPS, lFVII exhibits potent and general efficacy against a range of Gram-negative bacteria including XDR pathogens.k

### In vivo efficacy of lFVII against XDR *P. aeruginosa* and *A. baumannii* infections

**Fig. 6:**
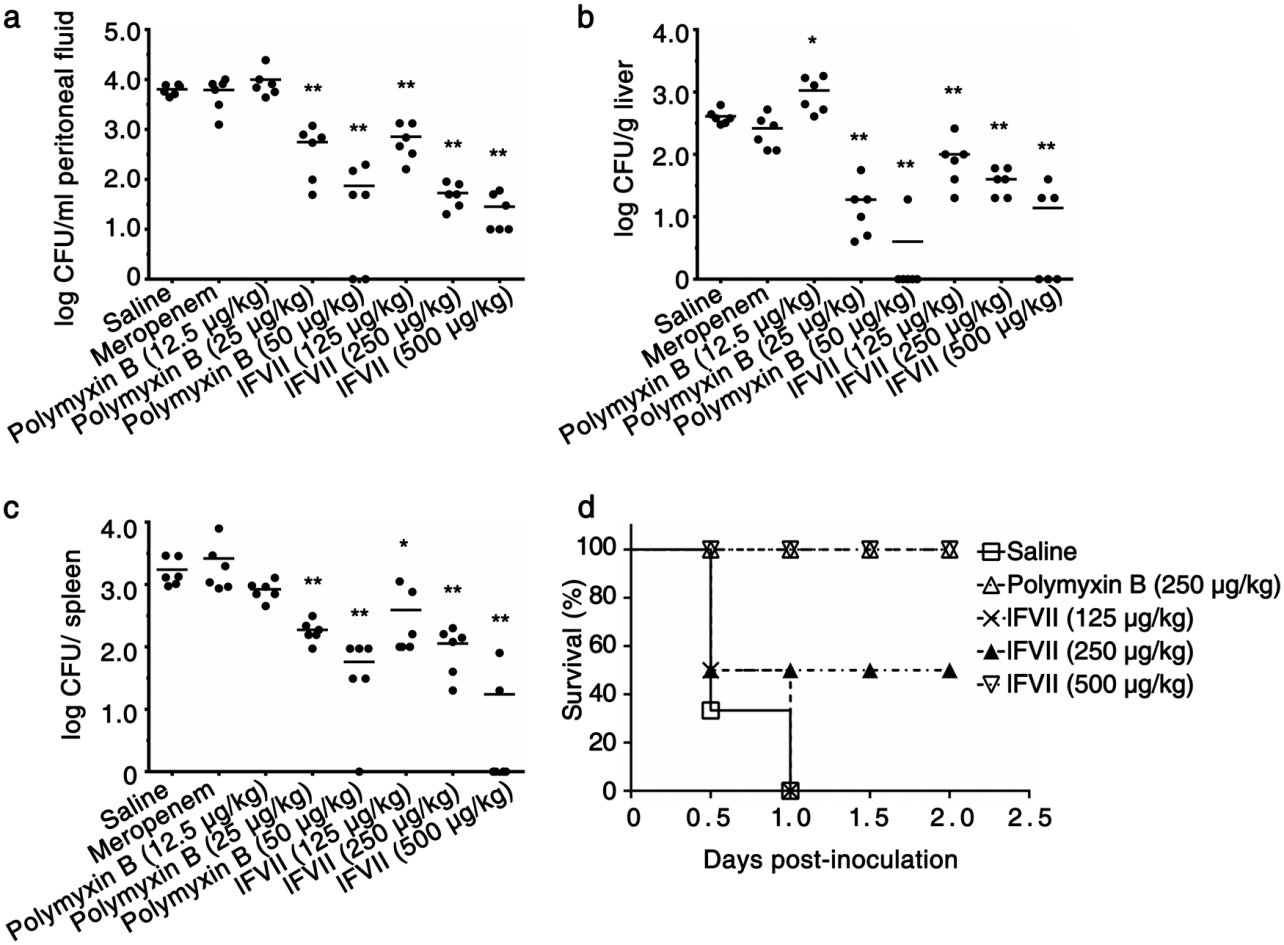
In vivo efficacy of the lFVII against XDR *P. aeruginosa* infection. **a**–**c** Decreased bacterial load in peritoneal fluid (**a**), liver (**b**), and spleen (**c**) by injecting lFVII. Thirty minutes after intraperitoneal inoculation of 7 × 10^7^ CFU of the XDR *P. aeruginosa* PA4, groups of six mice were injected intraperitoneally with saline, meropenem (2.5 mg/kg), polymyxin B, and lFVII. The dose of polymyxin B and lFVII were increased as indicated. Bacterial titers remaining in peritoneal fluid, liver and spleen were assayed at 24 h post injection of different agents. Bars indicate the means (*n* = 6). *P < 0.05 and **P < 0.01, significantly different from the saline-injected group. **d** Enhanced resistance to the XDR *P. aeruginosa* PA4 by injecting lFVII in mice. Thirty minutes after intraperitoneal inoculation of 9.0 × 10^7^ CFU of *P. aeruginosa* PA4, groups of six mice were injected intraperitoneally with saline, polymyxin B, and lFVII. The dose of lFVII was the same as those in **a**–**c**

The successful confirmation of the general antibacterial activity of lFVII against Gram-negative bacteria in vitro prompted assays for its in vivo efficacy against drug-resistant Gram-negative bacterial infections. Following intraperitoneal inoculation of the XDR *P. aeruginosa* PA4, BALB/c mice were intraperitoneally injected with different agents. Compared with the saline-treated groups, mice treated with lFVII presented reduced number of bacteria in peritoneal fluid and in liver and spleen tissues; furthermore, lFVII, as well as the positive control polymyxin B, decreased the bacterial cell numbers in a dose-dependent manner. Meropenem treatment had no effect on levels of the XDR bacteria as expected, and this was comparable to the saline-treated group (Fig. 6a–c). We next tested the ability of lFVII to protect mice against *P. aeruginosa* PA4 challenge at a lethal level. All the mice in saline-treated group died 24 h after bacterial inoculation, whereas the lFVII treatment improved the survival rate in a dose-dependent manner (Fig. 6d). Remarkably, all mice treated with the high dose of lFVII (500 µg/kg) survived with no signs of animal distress.

The effectiveness of lFVII against drug-resistant infection was also confirmed by using another XDR strain, *A. baumannii* Ab3, which along with the different agents were subjected to an intravenous instead of the intraperitoneal injection (Supplementary information, Fig. S8). The results were analyzed by taking into account the average blood volume of mice and the in vitro MBC values of agents. Relative to the positive control polymyxin B that function at ~26× in vitro MBC (250 µg/kg), lFVII exhibited a visible anti-infective effect at a low concentration that corresponds to ~1× in vitro MBC (250 µg/kg).

In this study, we noticed that, relative to XDR *A. baumannii* Ab3, XDR *P. aeruginosa* PA4 is more sensitive to the lFVII treatment in spleen (Fig. 6c and Supplementary information, Fig. S8c). This difference may result from the different bacterial strains or distinct injection methods.

## Discussion

Despite many efforts made to develop effective antibacterial agents, certain mammalian proteins with intrinsic antibacterial activity might be underappreciated. Here, we show that FVII, FIX and FX exert a direct antibacterial effect towards Gram-negative bacteria including XDR pathogens through LC-catalyzed LPS hydrolysis.

The well-established role of FVII, FIX, and FX is to initiate coagulation via their serine protease activity.^8–10^ Nevertheless, an involvement of these coagulation factors in anti-infection mechanisms of organisms has also been suggested because their deficiency is documented to have a significant correlation with bacterial infectious diseases such as sepsis and pneumonia.^18–20^ In the current study, we reveal their potent antibacterial activity against Gram-negative bacteria, proposing a concept that FVII, FIX and FX constitute a class of important antimicrobial proteins. Using FIX and lFIX as an example, our study suggests that while serine protease activity of the factors is resistant to heat treatment (Supplementary information, Fig. S9a),^35^ their antibacterial activity is thermo-sensitive (Supplementary information, Fig. S9b). This should be reasonable because the two different activities, as revealed by previous^8–10^ and the current studies, are attributed to two separate domains of the factors, HC and LC, respectively. On the other hand, it seems reasonable to assume that these factors might exhibit a lower level of antibacterial efficacy at their physiological concentrations (lower than their MICs that we determined), and that, in a case of injury, the recruitment of these factors to wound should cause an increased local concentration, which might facilitate their antibacterial function. However, the underlying details need further investigation.

After infection with pathogens, specialized recognition receptors identify specific pathogen-associated chemical moieties and then trigger activation of the innate immune system.^36^ Antimicrobial peptides are an integral part of the innate immune response and possess broad-spectrum antimicrobial activity.^37^ There is widespread acceptance that most naturally occurring peptides operate through their amphipathic structures, which enable binding and the following permeabilization or disruption of microbial cytoplasmic membranes.^38^ Accordingly, a class of antimicrobial peptide polymers, named SNAPPs, was recently designed to function via an electrostatic contact with the target bacteria.^32^ Alternatively, some antimicrobial peptides may penetrate into the cell and then interact with intracellular targets, leading to disruption of various key cellular processes such as cell envelope biosynthesis.^39^ Our analyses, however, clearly show that the antibacterial property of these coagulation factors is attributed to the LC-triggered LPS hydrolysis, presenting a new antimicrobial mechanism against Gram-negative bacteria. Moreover, it is likely that our finding is not an isolated case. FVII, FIX and FX exhibit a high conservation at the amino acid level (Supplementary information, Figs. S10, 11), suggesting that the antibacterial mechanism against Gram-negative bacteria reported here might be conserved through evolution and operate in the same manner in different vertebrates.

LPS is a major component of the outer membrane of Gram-negative bacteria, and is a critical player in the pathogenesis of infectious diseases caused by Gram-negative bacteria such as sepsis.^40^ Importantly, it is also crucial for the survival of Gram-negative bacteria.^13,14^ Thus, identification of LPS as the substrate of hydrolysis by lFVII suggests that the LC-induced cell envelope damage should happen to, and the antibacterial activity described here would be general for, any strains of Gram-negative bacteria. Consistently, antibacterial spectrum analysis showed that the recombinant lFVII exhibited activity at nanomolar concentrations against all the tested Gram-negative bacteria, including XDR strains; moreover, it also functioned in vivo, protecting animals against XDR Gram-negative bacterial infections.

According to a report,^41^ FX can activate innate immunity to human species C adenovirus through binding and decorating the entered virus particles. Interestingly, FX, as well as FIX, was also found to enhance the infection of adenovirus types 5 and 31,^42^ thereby giving rise to an adverse effect. Thus, a comprehensive investigation is needed for understanding the detailed role of FIX and FX in host defense against different viruses if they could be used as antibacterial agents. On the other hand, identifying the core functional domain, improving the antibacterial activity and reducing the unwanted side effects are also important for the development of antibacterial agents using these coagulation factors. We have revealed the EGF1 and EGF2 domains as the functional regions of lFVII. Moreover, several EGF1 mutants (e.g., C5G, L20G, G33A, etc.) were shown to have greater activity relative to the wild-type EGF1. They could be promising antimicrobial peptides against drug-resistant Gram-negative pathogens. However, the future study on an adverse effect of several EGF1 mutants is needed.

This study presents a new mechanism through which the coagulation factors FVII, FIX and FX act as antimicrobial proteins, and may advance our understanding of the coagulation system in host defense. Coagulation disorders are correlated with many diseases such as sepsis and stroke. Thus, this study also implies that these factors may participate in the pathogenesis of these diseases via their dual functions in blood coagulation and resistance to infection. On the other hand, our work may have broad prospects in clinical application. None of the known antibacterial agents has been reported to function by hydrolyzing LPS. Identification of the LPS hydrolysis-based antibacterial mechanism, combined with the antibacterial features described above and the characteristics associated with low-cost large-scale manufacturing, may offer new and cost-effective strategies for combating the urgent public health crisis posed by drug-resistant Gram-negative pathogens.

## Materials and methods

### Cell line, bacterial strain, and plasmid construction

Cell lines, bacterial strains, and plasmids used in the current study are listed in Supplementary information, Table S2. CHO-DG44 was obtained from Invitrogen (Cat# A1100001) and HepG2 was obtained from ATCC (HB-8065). The cell lines were mycoplasma free and passaged no more than 1 month after resuscitation. All cell lines were cultured according to the Invitrogen or ATCC instructions. *Escherichia coli* BL21(DE3) and DH5α were obtained from Invitrogen (Cat# C600003 and Cat# 18288019, respectively). *Acinetobacter baumannii*, *Klebsiella pneumoniae* and *Pseudomonas aeruginosa* were obtained from ATCC (19606, 4352 and 27853, respectively). All bacterial strains were cultured according to the ATCC instructions. The clinically isolated bacteria, including *A. baumannii* (Ab3, Ab5, Ab16, Ab18, Ab30, Ab35, Ab42, Ab46, Ab48), *Enterobacter cloacae* (Y1) and *P. aeruginosa* (L93, PA4, PA3), were collected from different departments (intensive care unit (ICU), gastroenterology, respiratory, neurosurgery and other wards) by the First Affiliated Hospital of Chengdu Medical College, Chengdu, Sichuan, China from 2012 to 2013.^33^ The isolates were identified by standard laboratory methods and ATB New (bioMérieux, France), and were further verified by PCR. All bacteria were grown on tryptose agar or Mueller–Hinton broth or agar (OXOID). Resistance of these isolates to different antibiotics was identified through dilution method described in the guidelines from the Clinical and Laboratory Standards Institute (CLSI, 2014), and the MIC was measured. The results were interpreted according to the CLSI guidelines (CLSI, 2014).

HepG2 cells were used for *FVII*, *FIX* and *FX* cloning; proteins studied in this paper were expressed in *E. coli* BL21 (DE3) or CHO-DG44 cells. *E. coli* BL21 (DE3) cells were cultured at 37 °C in LB medium; HepG2 and CHO-DG44 cells were cultured in DMEM containing 10% fetal bovine serum (FBS) and 1% penicillin-streptomycin in a 5% CO_2_ incubator at 37 °C. For protein production, cDNA fragments were generated using a standard or overlap PCR with specific primers containing suitable restriction sites (Supplementary information, Table S3). The PCR fragments and their dedicated plasmids were purified with DNA purification kits (Foregene), and then digested with the corresponding enzymes. The appropriate fragments were ligated. For *FVII*, *FIX* and *FX* cloning, cDNA fragments corresponding to the mature peptides were obtained from the total RNA of HepG2 cells using RT-PCR kit (Foregene). The DNA sequences encoding mCherry and recognition site of recombinant Tobacco Etch Virus protease (rTEV) were used for constructing the expression plasmids pET19-mCherry-rTEV-FVII-Gla, pET19-mCherry-rTEV- FVII-EGF1, pET19-mCherry-rTEV- FVII-EGF2 and pET19-mCherry-rTEV- FVII-EGF1mut, which were expected to facilitate production of the small-molecular-weight proteins. All of the cloned sequences were confirmed by DNA sequencing.

### Mice

Six-week-old male BALB/c mice obtained from the Laboratory Animal Academy of the Sichuan Medical Sciences Institute were maintained under standardized pathogen-free conditions at the animal care facility at State Key Laboratory of Biotherapy of Sichuan University. All mice weighted 20–22 g upon infection. At the end of this study, mice that were still alive were euthanized by CO_2_ exposure followed by cervical dislocation. All mouse experiments were reviewed and approved by the Animal Ethics Board and the Animal Care and Use Committee at the School of Life Sciences, Sichuan University (Project # 2019062801), and efforts were made to minimize suffering.

### Protein production

*E. coli* BL21 (DE3) was transformed with pET19-lFVII, pET19-lFIX, pET19-lFX, pET19-mCherry-rTEV-FVII-Gla, pET19-mCherry-rTEV-FVII-EGF1, pET19-mCherry-rTEV-FVII-EGF2 and pET19-mCherry-rTEV-FVII-EGF1mut, respectively. The transformed bacteria were grown in LB at 37 °C. At an OD_600_ of 0.6, IPTG was added to a final concentration of 1 mM, and the cells were grown overnight at 18 °C with shaking at 180 rpm. The resulting bacteria were collected by centrifugation and then broken by sonication in equilibration/wash buffer (EW buffer, 300 mM NaCl, 1% PMSF and 50 mM sodium phosphate, pH 7.4). Following 30 min of centrifugation at 40,000 × *g*, the His-tagged proteins in the supernatant were purified using HisPur™ Cobalt Resin (Thermo Fisher) following the manufacturer’s protocol. The purified proteins were dialyzed against dialysis buffer (150 mM NaCl, 25 mM Tris-HCl, pH 7.4) and then concentrated using an Amicon Ultra 10K device (Millipore).

For the stable eukaryotic expression of His-tagged hFVII, FVII, FIX and FX, CHO-DG44 cells were transfected with the plasmid pcDNA3.1-His-hFVII, pcDNA3.1-His-FVII, pcDNA3.1-His-FIX and pcDNA3.1-His-FX, respectively, using TransEasy reagent (Foregene). Two days post transfection, the transfected cells were selected with 800 µg/mL Geneticin (G418, GIBCO) for 10 days, and then maintained with 400 µg/mL G418. The cell clones were isolated, and the expression levels of His-tagged proteins were analyzed by western blot. The generated CHO-DG44-hFVII, CHO-DG44-FVII, CHO-DG44-FIX, and CHO-DG44-FX cells were cultured in DMEM supplemented with 10% FBS and 400 µg/mL G418. Once the culture reached 80% confluence, the medium was replaced with serum-free CHO medium (EX-CELL CD CHO, SAFC Biosciences). Three days later, the culture medium was collected by 5 min of centrifugation at 1000× *g*, and the His-tagged proteins in the supernatant were purified, dialyzed and concentrated as described above.

FVII was activated using a two-step method.^15^ Briefly, 300 nM FVII was incubated for 1.5 h at ambient temperature with 3 nM rFVIIa (Novo Nordisk) in a buffer solution (0.25 mM CaCl_2_, 100 mM NaCl, 1 µg/mL poly-D-lysine, 10 mM Tris-HCl, pH 7.4). The activation reaction was stopped by the addition of 5 mM CaCl_2_.

To prepare the three domains of lFVII (i.e., Gla, EGF1 and EGF2) and the EGF1 mutants, the purified fusion proteins His-mCherry-rTEV-FVII-Gla, His-mCherry-rTEV-FVII-EGF1, His-mCherry-rTEV-FVII-EGF2 and His-mCherry-rTEV-FVII-EGF1mut were treated with AcTEV (Invitrogen) to cleave their His-mCherry tags. The purified fusion proteins were collected on Ni-NTA agarose (QIAGEN) following 1-h incubation at 4 °C. The agarose was centrifuged at 700 × *g* for 3 min at 4 °C and then suspended in 450 µL of TBS buffer before treatment with 100 U of AcTEV, and the resulting mixture was incubated at 4 °C overnight. The supernatant containing the Gla, EGF1, EGF2 or EGF1mut was harvested by a 5-min centrifugation at 700 × *g* and 4 °C, and the protein samples were concentrated and purified by gel-filtration chromatography using a Superdex 75 5/150 GL column (GE Healthcare) on ÄKTA Explorer FPLC system (Amersham Pharmacia) with running buffer (50 mM Na_2_HPO_4_/NaH_2_PO_4_, 5 mM β-Mercaptoethanol and 300 mM NaCl, pH 7.4). The protein fractions were collected and concentrated with Amicon Ultra 3K device (Millipore).

### Western blot

The protein samples were fractionated with SDS-PAGE and electronically transferred onto PVDF membranes at 100 V over 1 h at 4 °C. The membranes were then rinsed with PBST buffer (0.2% Tween-20 in phosphate-buffered saline (PBS)), blocked for 2 h in PBST containing 5% non-fat milk powder, and incubated with a specific primary antibody for 1 h at ambient temperature. The membranes were then washed with PBST before incubation with horseradish peroxidase-conjugated IgG for 1 h. The resulting blots were visualized using ShinEasy Subpico ECL kit (Foregene) and exposed to X-ray film.

### Growth kinetics analysis

*E. coli* DH5α was grown in LB at 37 °C until the OD_600_ reached 0.6, and diluted 10-fold. One hundred microliter aliquots were then added to each well of microtiter plates containing pre-warmed LB supplemented with the LCs, antibacterial agents, or no drug (untreated). The plates were covered and incubated at 37 °C with shaking at 180 rpm for different incubation periods. The OD_600_ measurements were performed on a universal microplate spectrophotometer (BioTek). In addition, specific antibody was added along with FVII, FIX or FX to test the effect on the antibacterial activity of FVII, FIX, and FX.

### Killing kinetics analysis

*E. coli* DH5α was grown in LB overnight and diluted in fresh medium to an OD_600_ of 0.1. Following the addition of the LCs at concentrations of 4 × MIC (100 µg/mL), the bacteria were cultured at 37 °C with shaking at 180 rpm, and then collected at defined intervals. The bacteria collected were diluted in PBS, and 100 µL samples of the dilutions were spreaded onto LB agar plates. The plates were incubated at 37 °C for 16 h, and the resulting CFUs were counted.

### Loss-of-function assay

Neutralization of the endogenous FVII, FIX or FX was carried out by intravenous injection of the specific antibodies. Commercial antibody products, including anti-mFVII antibody (R&D Systems), anti-mFIX antibody (K-20, Santa Cruz Biotechnology), anti-mFX antibody (C-20, Santa Cruz Biotechnology), normal rabbit IgG (Santa Cruz Biotechnology) and normal goat IgG (Santa Cruz Biotechnology) were diluted with saline to prepare the antibody solutions as follows: solution 1, containing 10 μg/mL of anti-mFVII antibody; solution 2, containing 80 μg/mL of anti-mFIX antibody; solution 3, containing 80 μg/mL of anti-mFX antibody; solution 4, containing all of the antibodies specific for mFVII, mFIX and mFX with the concentrations described above; solution 5, containing 160 μg/mL of normal rabbit IgG; solution 6, containing 160 μg/mL of normal goat IgG. Solutions 1–4 were used to neutralize the endogenous FVII, FIX and FX, and solutions 5 and 6 were used as the antibody controls. Male BALB/c mice (6 weeks old, weighing 20–22 g) were injected via tail vein with 0.1 mL of the prepared antibody solutions (for each solution injection, *n* = 8). After 1 h of the antibody injection, mice were inoculated via tail vein with 1 × 10^6^ CFU/mL of bacteria in 0.2 mL saline. The number of surviving animals at various time points was counted. Kaplan–Meier analysis was used to determine the significance of the differences between the experimental and control groups. In addition, after 3 h of the antibody injection, blood was collected from the retro-orbital sinus, and sodium citrate was added to a final concentration of 10.9 mM. The blood samples were centrifuged at 3000 rpm for 15 min, and the separated plasma samples were used to determine the aPTT and PT with a hemagglutination analyzer (KING DIAGNOSTIC).

### Bacterial load in blood, peritoneal fluid, liver and spleen

The bacterial load in blood, peritoneal fluid, liver or spleen of mice was measured as described.^17,32^ After 2 h of bacterial inoculation, blood was collected from the retro-orbital sinus and immediately mixed with heparin, peritoneal fluid was recovered from the peritoneum, and liver as well as spleen tissues were harvested from killed mice, weighed, and homogenized in saline. Serial dilutions of the blood, peritoneal fluid and tissue homogenates were plated on selective agar plates and incubated overnight at 37 °C. Cetrimide selective agar (OXOID) was used for the culture of *P. aeruginosa*, and acinetobacter selective agar (CHROMagar) was used for *A. baumannii* growth. CFUs were calculated as CFU/mL for blood or peritoneal fluid and as CFU/g of wet weight for liver tissues. Since the weight of the spleen in mice is <1 g, the CFUs for spleen were calculated as CFU per fresh organ.

### Antibacterial activity of the LCs against starved *E. coli* DH5α

After overnight growth in LB at 37 °C, *E. coli* DH5α cells were washed and diluted in hypertonic TBS (TBS buffer containing 15% (w/v) sucrose) to an OD_600_ of 0.1. After the addition of different antibacterial agents, bacteria were incubated at 37 °C for 4 h with shaking at 180 rpm, and then diluted with hypertonic TBS. The dilutions were plated onto hypertonic LB agar plates and incubated at 37 °C for 16 h. The final number of CFUs was counted. The same experiment was repeated with hypotonic TBS (TBS buffer containing 0.1% (w/v) NaCl) and hypotonic LB agar plates.

### SEM detection

*E. coli* DH5α was grown in LB to an OD_600_ of 0.2. Following the addition of the LCs, lysozyme, polymyxin B, or no drug (untreated), the bacteria were incubated at 37 °C with shaking at 180 rpm and harvested at different time points (1.5, 2.5 and 4 h). The incubation was also conducted for the purified PGNs of *E. coli* K12, *S. aureus* and *B. subtilis* (Invivogen) in PBS buffer for 3 h. The bacteria or PGNs harvested were washed before being resuspended in PBS buffer for SEM detection.

The treated bacterial and PGN samples were dropped on slides and the slides were placed on a hot plate at 37 °C to dry. The slides then were soaked in 2% glutaraldehyde in PBS for 2 h, before thorough wash with PBS buffer. The samples fixed on the slides were dehydrated through an ascending alcohol series (30%, 50%, 70%, 80%, 90 and 100% EtOH) for 15 min each, followed by a 15-min soak in 100% EtOH. Following critical point drying, the samples were visualized by SEM.^43^

### Interactions of the LCs with LPS

The interactions were verified through 3 major procedures: (1) preparation of DPX; (2) DPX binding experiments; and (3) binding inhibition experiments.

Preparation of DPX was performed as described^44^ and is detailed as follows. A solution of 10 mg of dansyl chloride (Sigma-Aldrich) in acetone (0.8 mL) was incubated with 40 mg of polymyxin B-sulfate (Sigma-Aldrich) in a 0.1 M solution of NaHCO_3_ (1.2 mL) without stirring and in the absence of light for 90 min at ambient temperature. The supernatant was applied to a PD-10 column (Sephadex G25M, GE Healthcare) and eluted with 0.145 M NaCl in a 0.01 M PBS buffer (pH 7.4). A peak showing orange fluorescence under ultraviolet light was collected and found to contain the DPX. The derivative, DPX, was extracted from the pooled fractions with n-butanol. Subsequent removal of the solvent by vacuum freeze drying gave the purified DPX as a pale yellow powder. The amount of DPX was determined by dinitrophenylation.

After DPX preparation, the DPX binding experiments were carried out to determine the optimal concentration of DPX. The fluorescence of the LPS-binding DPX was measured using a spectral scanning multimode reader (Thermo Fisher) at an excitation wavelength of 325 nm and an emission wavelength of 554 nm. The binding assays were performed by measuring the fluorescence in the wells following the addition of portions of DPX to the wells of the microtiter plate containing 0.3 µg LPS in 100 µL of 5 mM HEPES buffer (pH 7.4). In this study, the optimal concentration of DPX was determined to be 3 µM.

In the binding inhibition experiments, the LCs and polymyxin B, which were used as completive inhibitors of the binding of DPX to LPS, were titrated into the wells of the microtiter plate containing 0.3 µg of LPS in 100 µL of 5 mM HEPES buffer (pH 7.4). After 30 min of incubation at 37 °C, DPX was added to a final concentration of 3 µM. The reduction in fluorescence (percent inhibition) was recorded. BSA was used as a negative control for the LCs.

### Sample preparation for MS analysis

Sixty micrograms of lFVII was mixed with 250 µg of *E. coli* K12 LPS (Invivogen) or 280 µg of *E. coli* F583 lipid A (Sigma-Aldrich) in 400 µL of saline, and the resulting mixture was incubated overnight at 37 °C with shaking at 160 rpm. The supernatant of reaction mixture was then collected following 10 min of centrifugation at 17,000 × *g* before dialysis against distilled water. The purified lFVII and lFVII-untreated *E. coli* K12 LPS or *E. coli* F583 lipid A were prepared as controls.

### MALDI/TOF-MS

One microliter of a 10 mg/mL solution of 2,5-dihydroxybenzoic acid (DHB) in 33% ethanol was applied to a MTP 384 ground steel TF target plate (Bruker Daltonics). The 1 µL sample prepared above was then mixed into the DHB droplet and dried under a stream of air. The samples were analyzed with an Autoflex TOF/TOFII instrument (Bruker) with a nitrogen 377 nm laser beam. The instrument was operated in positive acquisition mode and controlled using the FlexControl 2.2 software package. All of the spectra were obtained using the reflector mode with an acceleration voltage of 19 kV, a reflector voltage of 20 kV, and pulsed ion extraction of 140 ns in the positive ion mode over an acquisition range (*m/z*) of 600–7000. The data were collected from an average of 500 laser shots, using the lowest laser energy necessary to obtain sufficient signal to noise ratios. Peak lists were generated from the MS spectra using the Bruker Flex Analysis software (Version 3.0). Post-source decay spectra using the Bruker Daltonics LIFT system were recorded at precursor ion acceleration and fragment acceleration voltage of 18.96 kV (LIFT voltage 4.37 kV). The reflector voltages 1 and 2 were set to 23.49 and 9.69 kV, respectively. The samples were also analyzed by no-matrix MALDI-TOF-MS over an acquisition range (*m/z*) of 100–2000 in positive or negative ion mode.^45^

### ESI-MS

ESI-MS analyses were performed on a Micromass Q-TOF premier mass spectrometer (Waters) in positive or negative ion mode. The samples were infused through the capillary head at 5 kV into the ion source using a linear syringe pump at a rate of 10 µL/min, and the spectra were scanned over an acquisition range (*m/z*) of 100–2000. Nitrogen was used as the curtain gas.^46^

### Kinetic analysis of lFVII activity towards *E. coli* K12 LPS

Assays using *E. coli* K12 LPS as the substrate were performed in triplicate, and the *E. coli* K12 LPS contained in reaction mixtures was color developed using Chromogenic End-point Tachypleus Amebocyte Lysate (CETAL, Xiamen Bioendo Technology, Co., Ltd, China) and measured using a universal microplate spectrophotometer (BioTek) at 545 nm. The range of substrate concentrations used for determining *Km* and *Vmax* values was 0.04–2.6 mM. For the assay, *E. coli* K12 LPS prepared in 10 µL of saline at 2× its final concentration was added to 10 µL of saline containing 1 µg of lFVII, and then the reactions were conducted at 37 °C for 1 h. At the beginning and the end of the reaction process, 5-µL aliquots were collected from reaction mixtures. After 10 min of heat inactivation at 70 °C, the collected samples were diluted appropriately to reach the linear range of CETAL. The subsequent color development and absorbance measurement were performed following the manufacturer’s protocol. Goodness-of-fit statistical analysis of the linear trendline of the resulting Lineweaver–Burk plot yielded an *r*^2^ value of 0.994.

*E. coli* K12 LPS was also used to determine temperature and pH activity profiles of lFVII. All reaction mixtures contained 1 µg of lFVII and 0.86 mM *E. coli* K12 LPS, and all reactions were performed in 20 µL volumes. To measure the optimum PH, a series of standard pH saline solutions over a range of pH 6.2–8.6 were used as the enzyme reaction buffer. To measure the optimum temperature, detailed studies were performed over a temperature range of 27–47 °C. The absorbance at 545 nm was measured using the same methods described above to determine the relative activity across the tested temperature and pH ranges.

### Analysis of LPS degradation by electrophoresis

*E. coli* K12 LPS (Invivogen), *E. coli* 055:B5 LPS (Sigma-Aldrich), or *A. baumannii* Ab3 LPS and *P. aeruginosa* PA4 LPS purified via a method of hot aqueous-phenol extraction^47^ was mixed with lFVII, lFIX, lFX, EGF1 or EGF1 mutant in saline, and the resulting mixtures were incubated at 37 °C with shaking at 160 rpm. After the incubation, supernatant of the reaction mixtures was collected by centrifugation at 17,000 × *g*, and then subjected to a Tricine-SDS-PAGE analysis as described.^48^

### NMR chemical shift perturbation experiments

A standard ^1^H–^13^C HSQC spectrum was recorded using a natural abundance of EGF1 sample (~3 mg/mL) on a 500 MHz Bruker NMR spectrometer (AVANCE III) at 298 K. Subsequently, two HSQC spectra were collected by adding LPS to the sample, with their molar ratio (EGF1:LPS) at 1:0.1 and 1:0.25. NMR data were processed using NMRPipe and analyzed with Sparky.

### Measurement of MBC

A dilution series of each antibacterial agent was made by diluting test agent stock in media to a desired range of concentrations and a final volume of 100 µL in each well of a microtiter plate. Bacterial cells, which gave an OD_600_ of 0.6, were diluted to 5 × 10^6^ cells/mL in media, and 100 µL of the bacteria solution was added to each well. The microtiter plate was then incubated at 37 °C for 3 h with shaking at 180 rpm. For each well, microbial solution was diluted with saline using an appropriate dilution factor and placed on an agar plate. The plates were incubated at 37 °C for 16 h, and the resulting CFUs were then counted and expressed as CFU/mL. The controls consisting of cells without any treatment were also measured. Concentration-killing curves were plotted with CFU/mL as a function of agent concentration, and linear regression analysis was used to determine the MBC values at which the CFU/mL becomes zero. For all of the tested bacteria, a nutritionally rich medium (MHB, OXOID) and TBS were used for the MBC measurement, respectively.

### Isolation of LPS-deficient *A. baumannii*

The LPS-deficient *A. baumannii* was isolated as described.^49^ In brief, an OD_600_ of 1.0 of *A. baumannii* Ab3 was plated on LB agar containing 10 μg/mL of colistin (Sigma-Aldrich). Isolated colonies were picked and replica plated on LB agar containing vancomycin (Sigma-Aldrich, 10 μg/mL) and LB agar containing colistin (10 μg/mL). Colonies sensitive to vancomycin, but resistant to colistin were deemed LPS-deficient.

### Statistical analysis

Student’s *t*-test was performed to compare the differences between treated groups and their paired controls. Data of bacterial loads were analyzed using a one-way classification analysis of variance (ANOVA) and Student’s *t*-test. All results were presented as the means ± SD and *P*-values < 0.05 were considered significant.

## Supplementary information

Supplementary information, Figure S1

Supplementary information, Figure S2

Supplementary information, Figure S3

Supplementary information, Figure S4

Supplementary information, Figure S5

Supplementary information, Figure S6

Supplementary information, Figure S7

Supplementary information, Figure S8

Supplementary information, Figure S9

Supplementary information, Figure S10

Supplementary information, Figure S11

Supplementary information, Table S1

Supplementary information, Table S2

Supplementary information, Table S3
