# Supplementary material for: Coagulation factors VII, IX and X are effective antibacterial proteins against drug-resistant Gram-negative bacteria
Source: Cell Res. 2019 Aug 9;29(9):711–24. doi: 10.1038/s41422-019-0202-3 (PMC6796875; doi:10.1038/s41422-019-0202-3)
Supplement: Supplementary file 1 — Supplementary information, Figure S1 [file 41422_2019_202_MOESM1_ESM.pdf]

Supplementary information, Figure S1

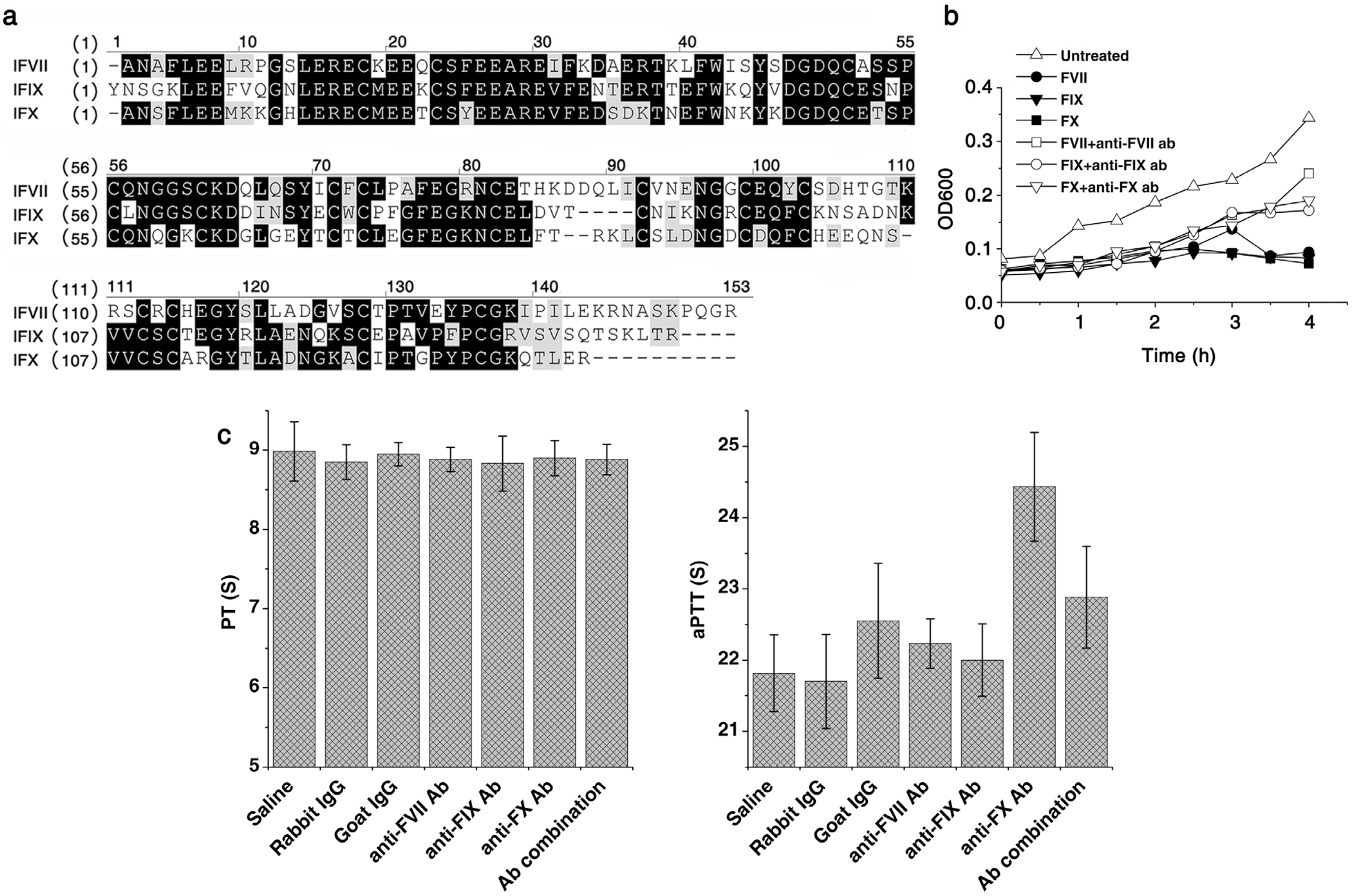

Fig. S1 FVII, FIX, and FX exhibit antibacterial activity. **a** Sequence alignments of the LC of FVII with the homologous FIX and FX. Sequence alignment was carried out using Vector NTI Advance 11 (Invitrogen). **b** Growth kinetic measurement of *E. coli* DH5α exposed to FVII (100 μg/ml), FIX (100 μg/ml) and FX (200 μg/ml) without or with addition of specific antibody (anti-FVII Ab, 100 μg/ml; anti-FIX Ab, 100 μg/ml; anti-FX Ab, 200 μg/ml). Untreated bacteria were included. Data represent mean values from five independent experiments. **c** PT (left) and aPTT (right) of the antibody-infused mice. The following antibodies (dose per mouse in parentheses), with saline as a blank control, were investigated in groups of eight mice: anti-FVII Ab (1 μg), anti-FIX Ab (8 μg), anti-FX Ab (8 μg), Ab combination (1 μg of anti-FVII Ab, 8 μg of anti-FIX Ab and 8 μg of anti-FX Ab), normal goat IgG (16 μg) and normal rabbit IgG (16 μg). PT and aPTT were determined after 3 h of the antibody infusion.
