# Supplementary material for: Coagulation factors VII, IX and X are effective antibacterial proteins against drug-resistant Gram-negative bacteria
Source: Cell Res. 2019 Aug 9;29(9):711–24. doi: 10.1038/s41422-019-0202-3 (PMC6796875; doi:10.1038/s41422-019-0202-3)
Supplement: Supplementary file 2 — Supplementary information, Figure S2 [file 41422_2019_202_MOESM2_ESM.pdf]

# Supplementary information, Figure S2

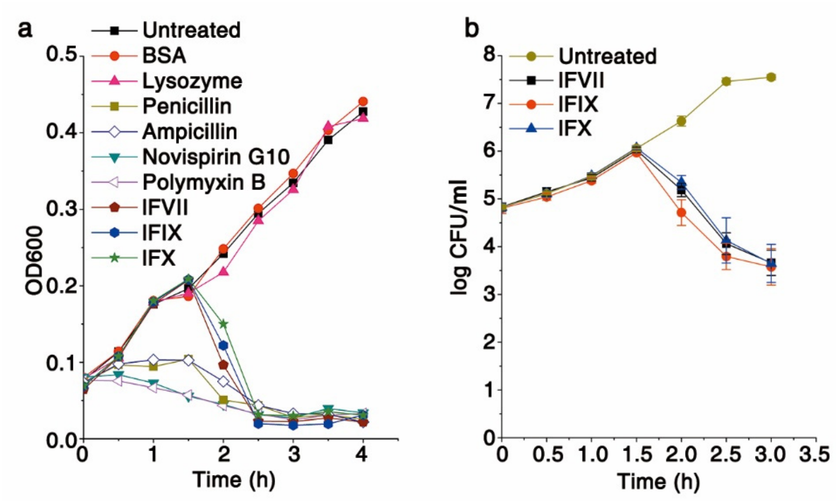


**Fig. S2** Antibacterial effects of the LCs towards *E. coli* DH5α. **a** Growth kinetic measurements of *E. coli* DH5α exposed to the LCs (4× MIC), BSA and other antibiotics with known cellular targets placed the LCs into the cluster of cell envelope-targeting agents. Data represent mean values from four independent experiments. **b** Killing kinetics of the LCs. The kinetic cell death of *E. coli* DH5α in LB was detected following its treatment with the LCs (4× MIC). Error bars represent SD (n=4). Untreated bacteria were included in all experiments. The final concentrations of different agents are listed as follows: BSA, 100 μg/ml; lysozyme, 50 μg/ml; penicillin, 100 μg/ml; ampicillin, 250 μg/ml; novispirin G10, 10 μg/ml; polymyxin B, 10 μg/ml; lFVII, 100 μg/ml; lFIX, 100 μg/ml; lFX, 100 μg/ml.
