# Supplementary material for: Coagulation factors VII, IX and X are effective antibacterial proteins against drug-resistant Gram-negative bacteria
Source: Cell Res. 2019 Aug 9;29(9):711–24. doi: 10.1038/s41422-019-0202-3 (PMC6796875; doi:10.1038/s41422-019-0202-3)
Supplement: Supplementary file 3 — Supplementary information, Figure S3 [file 41422_2019_202_MOESM3_ESM.pdf]

Supplementary information, Figure S3

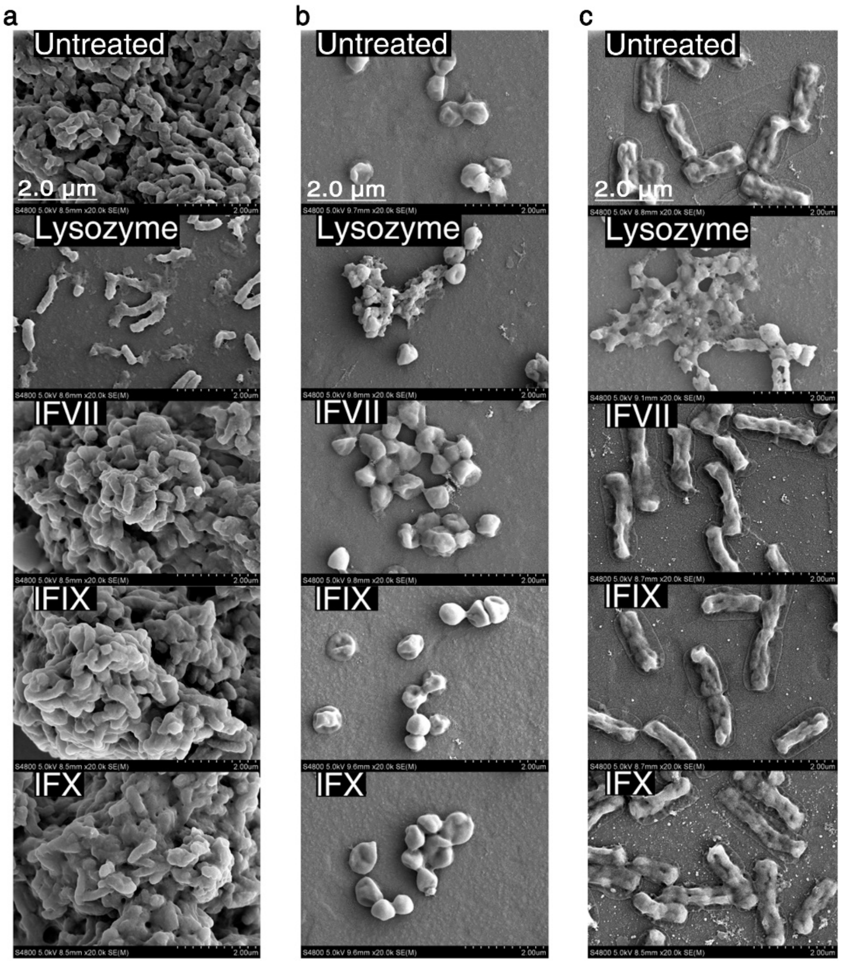

Fig. S3 LCs do not cause the degradation of PGN. PGN of *E. coli* K12 (**a**), *Staphylococcus aureus* (**b**) and *Bacillus subtilis* (**c**) in TBS were incubated for 3 h at 37 °C with the LCs or lysozyme, washed with TBS, and then scanned by SEM. Untreated PGN was included in all experiments. The final concentrations of the LCs and lysozyme are listed as follows: lysozyme, 50 μg/ml; lFVII, 100 μg/ml; lFIX, 100 μg/ml; lFX, 100 μg/ml.
