# Supplementary material for: Coagulation factors VII, IX and X are effective antibacterial proteins against drug-resistant Gram-negative bacteria
Source: Cell Res. 2019 Aug 9;29(9):711–24. doi: 10.1038/s41422-019-0202-3 (PMC6796875; doi:10.1038/s41422-019-0202-3)
Supplement: Supplementary file 4 — Supplementary information, Figure S4 [file 41422_2019_202_MOESM4_ESM.pdf]

Supplementary information, Figure S4

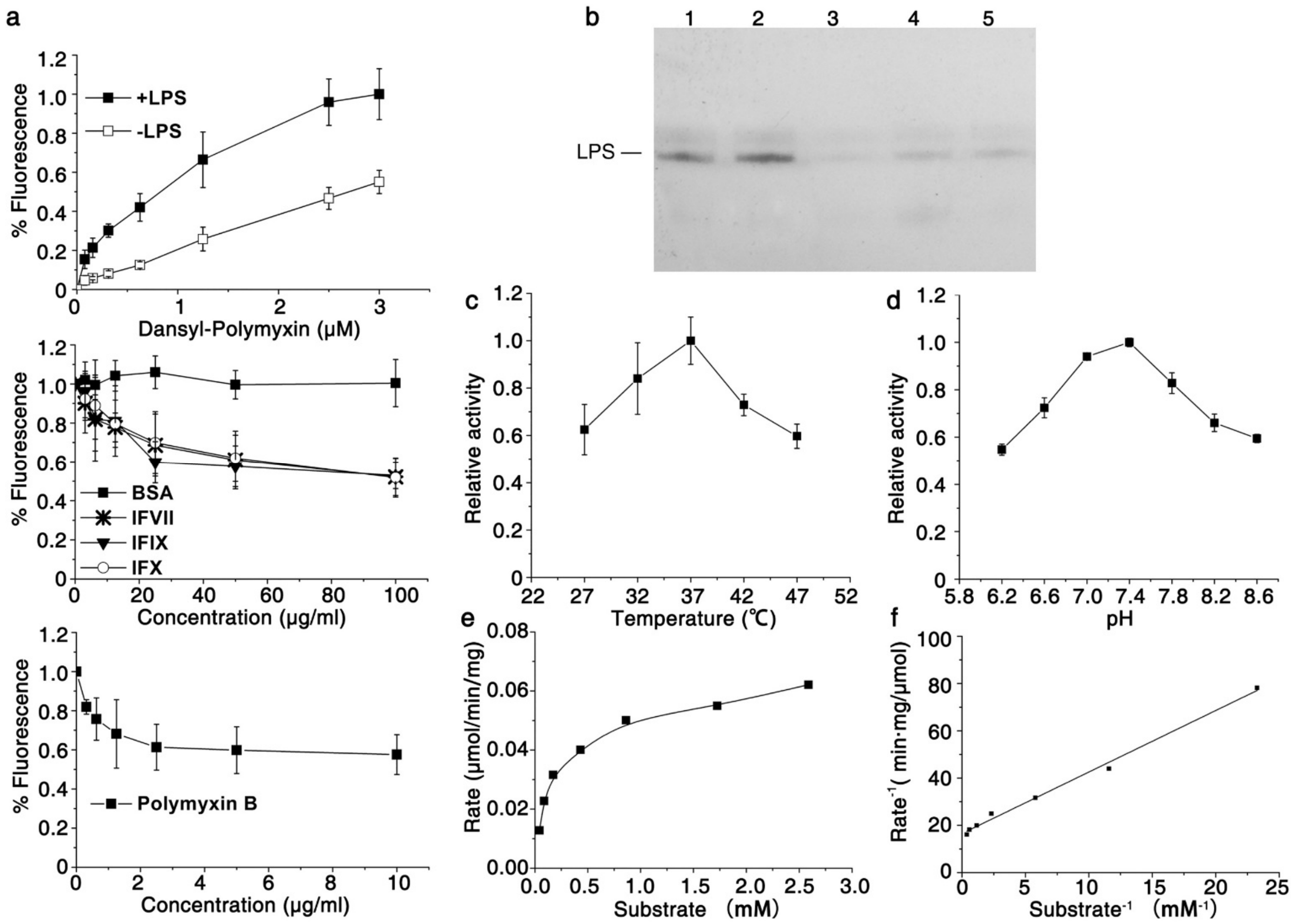

Fig. S4 LCs trigger the hydrolysis of *E. coli* K12 LPS. **a** The LC-dependent inhibition of DPX binding to LPS. DPX was titrated into HEPES buffer containing 0.3 μg of LPS (+LPS) or HEPES buffer alone (-LPS). The optimal concentration of DPX was determined to be 3 μM (top). Serial concentrations of the LCs or BSA were incubated for 30 min with 0.3 μg of LPS, and then DPX was added to a final concentration of 3 μM (middle). Polymyxin B was used as a positive control (bottom). The error bars represent SD (n=3). **b** LC treatments lead to a degradation of *E. coli* K12 LPS. Separated samples of untreated LPS (lane 1), BSA-treated LPS (lane 2), lFVII-treated LPS (lane 3), lFIX-treated LPS (lane 4), and lFX-treated LPS (lane 5) on Tricine-SDS-PAGE were examined by silver staining. **c, d** Activity of lFVII with *E. coli* K12 LPS. *E. coli* K12 LPS was used to determine temperature (**c**) and pH (**d**) activity profiles of lFVII. **e** Initial velocity of lFVII-catalyzed hydrolysis of *E. coli* K12 LPS. **f** Lineweaver-Burk plot of lFVII hydrolysis of *E. coli* K12 LPS. In **e** and **f**, hydrolysis reactions were performed under the optimum conditions of 37 °C and pH 7.4.
