# Supplementary material for: Coagulation factors VII, IX and X are effective antibacterial proteins against drug-resistant Gram-negative bacteria
Source: Cell Res. 2019 Aug 9;29(9):711–24. doi: 10.1038/s41422-019-0202-3 (PMC6796875; doi:10.1038/s41422-019-0202-3)
Supplement: Supplementary file 5 — Supplementary information, Figure S5 [file 41422_2019_202_MOESM5_ESM.pdf]

Supplementary information, Figure S5

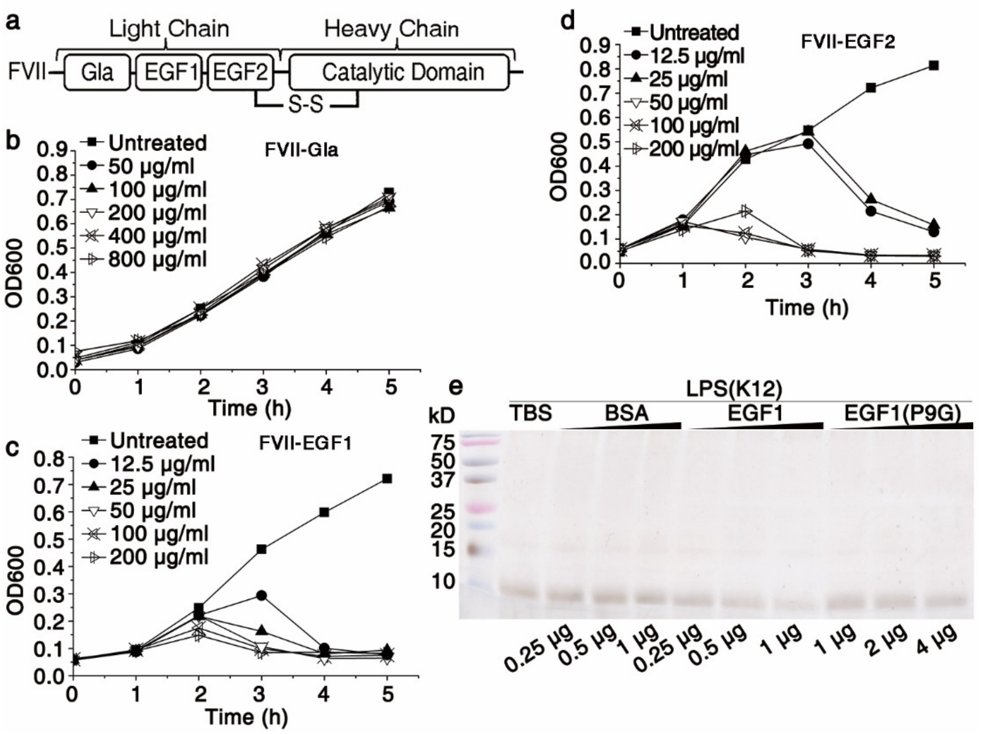

Fig. S5 The EGF-like domains of FVII exert antibacterial effect. **a** Diagrammatic structure of FVII. **b-d** Growth kinetics of *E. coli* DH5α exposed to different concentrations of FVII-Gla (**b**), FVII-EGF1 (**c**) and FVII-EGF2 (**d**). Data represent mean values from five independent experiments. **e** P9G mutation decreases the ability of EGF1 to hydrolyze *E. coli* K12 LPS. Twenty micrograms of *E. coli* K12 LPS was treated at 37 °C for 2 h per reaction; the dose of protein was increased as indicated; relative to wild-type EGF1, the P9G mutant was analyzed at 4× higher protein concentrations. Separated LPS samples on Tricine-SDS-PAGE were examined by silver staining.
