# Supplementary material for: Coagulation factors VII, IX and X are effective antibacterial proteins against drug-resistant Gram-negative bacteria
Source: Cell Res. 2019 Aug 9;29(9):711–24. doi: 10.1038/s41422-019-0202-3 (PMC6796875; doi:10.1038/s41422-019-0202-3)
Supplement: Supplementary file 6 — Supplementary information, Figure S6 [file 41422_2019_202_MOESM6_ESM.pdf]

Supplementary information, Figure S6

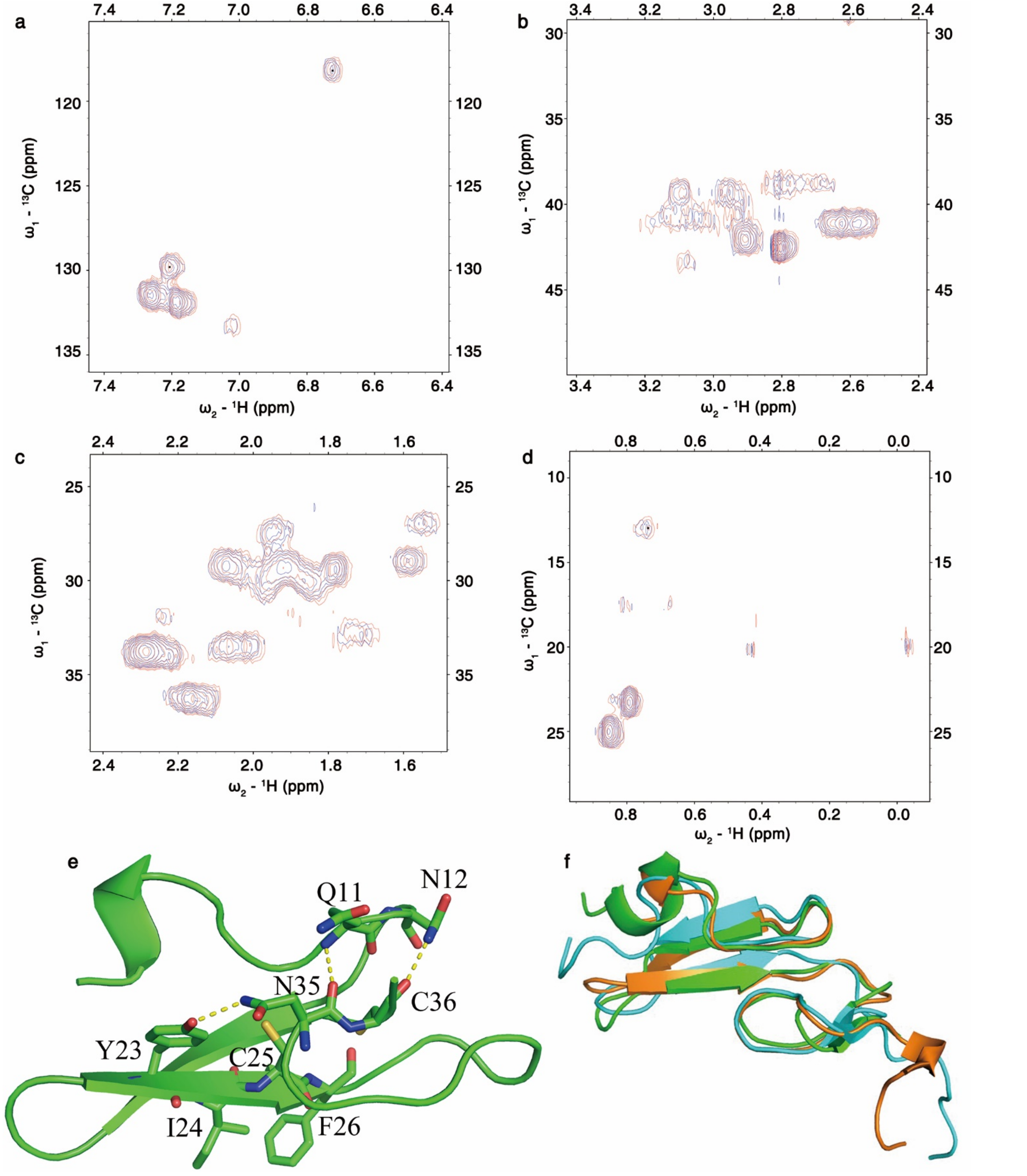

Fig. S6 Residues critical for the antibacterial activity of lFVII. **a-d** NMR titration spectra corresponding to the regions of side-chains of tyrosine and phenylalanine (**a**), asparagine and cysteine (**b**), glutamine (**c**) and isoleucine (**d**) were enlarged, respectively. **e** The hydrogen bonds network involving sequences $Q_{11}N_{12}$, $Y_{23}I_{24}C_{25}F_{26}$ and $N_{35}C_{36}$. **f** Structure comparison of EGF1 domain of FVII in crystal (PDB ID: 1QFK, orange) and solution (PDB ID: 1BF9, green; PDB: 1F7M, cyan).
