# Supplementary material for: Coagulation factors VII, IX and X are effective antibacterial proteins against drug-resistant Gram-negative bacteria
Source: Cell Res. 2019 Aug 9;29(9):711–24. doi: 10.1038/s41422-019-0202-3 (PMC6796875; doi:10.1038/s41422-019-0202-3)
Supplement: Supplementary file 7 — Supplementary information, Figure S7 [file 41422_2019_202_MOESM7_ESM.pdf]

Supplementary information, Figure S7

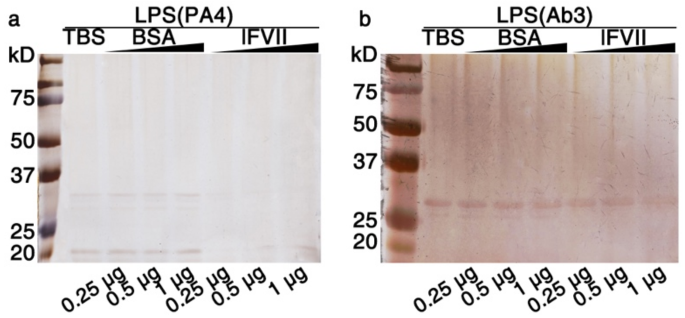

Fig. S7 lFVII degrades the LPS from XDR strains *P. aeruginosa* PA4 (**a**) and *A. baumannii* Ab3 (**b**). Ten micrograms of *P. aeruginosa* PA4 LPS or *A. baumannii* Ab3 LPS was treated at 37 °C for 2 h per reaction; the dose of protein was increased as indicated. Separated LPS samples on Tricine-SDS-PAGE were examined by silver staining.
