# Supplementary material for: Coagulation factors VII, IX and X are effective antibacterial proteins against drug-resistant Gram-negative bacteria
Source: Cell Res. 2019 Aug 9;29(9):711–24. doi: 10.1038/s41422-019-0202-3 (PMC6796875; doi:10.1038/s41422-019-0202-3)
Supplement: Supplementary file 8 — Supplementary information, Figure S8 [file 41422_2019_202_MOESM8_ESM.pdf]

Supplementary information, Figure S8

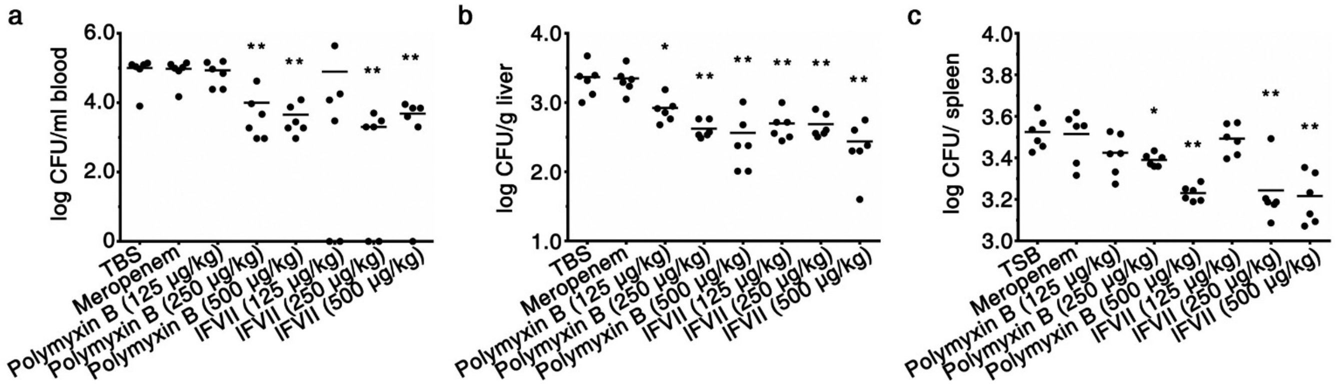

Fig. S8 *In vivo* efficacy of the lFVII against XDR *A. baumannii* infection. Thirty minutes after intravenous inoculation of 2×10^8 CFU of the XDR *A. baumannii* Ab3, groups of six mice were injected intravenously with saline, meropenem (40 mg/kg), polymyxin B, and lFVII. The dose of polymyxin B and lFVII were increased as indicated. Bacterial titers remaining in blood (**a**), liver (**b**), and spleen (**c**) were assayed at 24 h post injection of different agents. Bars indicate the mean values (n=6). *$P < 0.05$ and **$P < 0.01$, significantly different from the saline-injected group.
