# Supplementary material for: Coagulation factors VII, IX and X are effective antibacterial proteins against drug-resistant Gram-negative bacteria
Source: Cell Res. 2019 Aug 9;29(9):711–24. doi: 10.1038/s41422-019-0202-3 (PMC6796875; doi:10.1038/s41422-019-0202-3)
Supplement: Supplementary file 9 — Supplementary information, Figure S9 [file 41422_2019_202_MOESM9_ESM.pdf]

Supplementary information, Figure S9

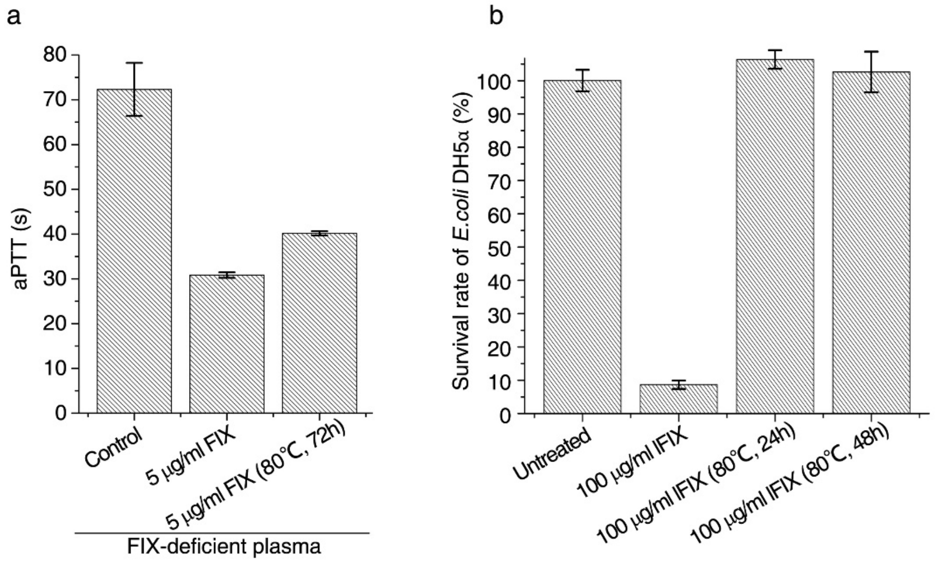

Fig. S9 Influence of heat treatment on the coagulant and antibacterial activity of FIX or lFIX. Heat treatment is one of the major methods for viral inactivation in the manufacturing process of plasma-derived coagulation factor products[35]. FIX was first subjected to a heat treatment at 80 °C for 72 h, and the treatment was shown to have only a slight impact on the ability of FIX to decrease the aPTT of FIX-deficient plasma **(a)**. Next, lFIX was treated at 80 °C for a shorter time (24 or 48 h), and its antibacterial activity was significantly impaired in these cases **(b)**. In **a**, FIX was tested at its physiological concentration in human (5 μg/mL); in **b**, lFIX was tested at the concentration of 100 μg/mL (4× MIC against *E. coli* DH5α determined in this study). Error bars represent SD (n=3).
