# Supplementary material for: Coagulation factors VII, IX and X are effective antibacterial proteins against drug-resistant Gram-negative bacteria
Source: Cell Res. 2019 Aug 9;29(9):711–24. doi: 10.1038/s41422-019-0202-3 (PMC6796875; doi:10.1038/s41422-019-0202-3)
Supplement: Supplementary file 10 — Supplementary information, Figure S10 [file 41422_2019_202_MOESM10_ESM.pdf]

# Supplementary information, Figure S10

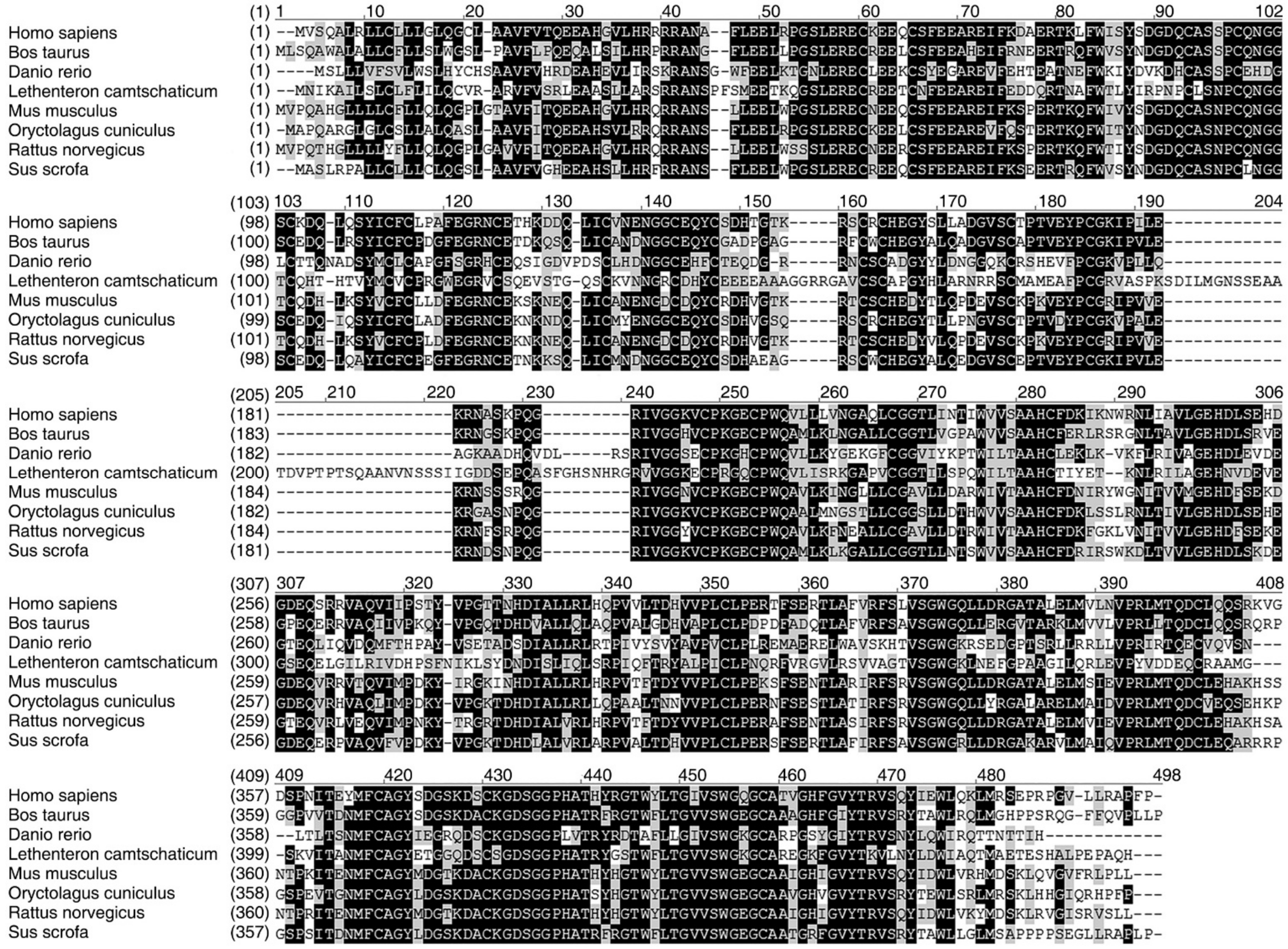


**Fig. S10** Conservation of FVII in different vertebrates. Sequence alignment was carried out using Vector Advance 11 (Invitrogen).
