# Supplementary material for: Coagulation factors VII, IX and X are effective antibacterial proteins against drug-resistant Gram-negative bacteria
Source: Cell Res. 2019 Aug 9;29(9):711–24. doi: 10.1038/s41422-019-0202-3 (PMC6796875; doi:10.1038/s41422-019-0202-3)
Supplement: Supplementary file 11 — Supplementary information, Figure S11 [file 41422_2019_202_MOESM11_ESM.pdf]

Supplementary information, Figure S11

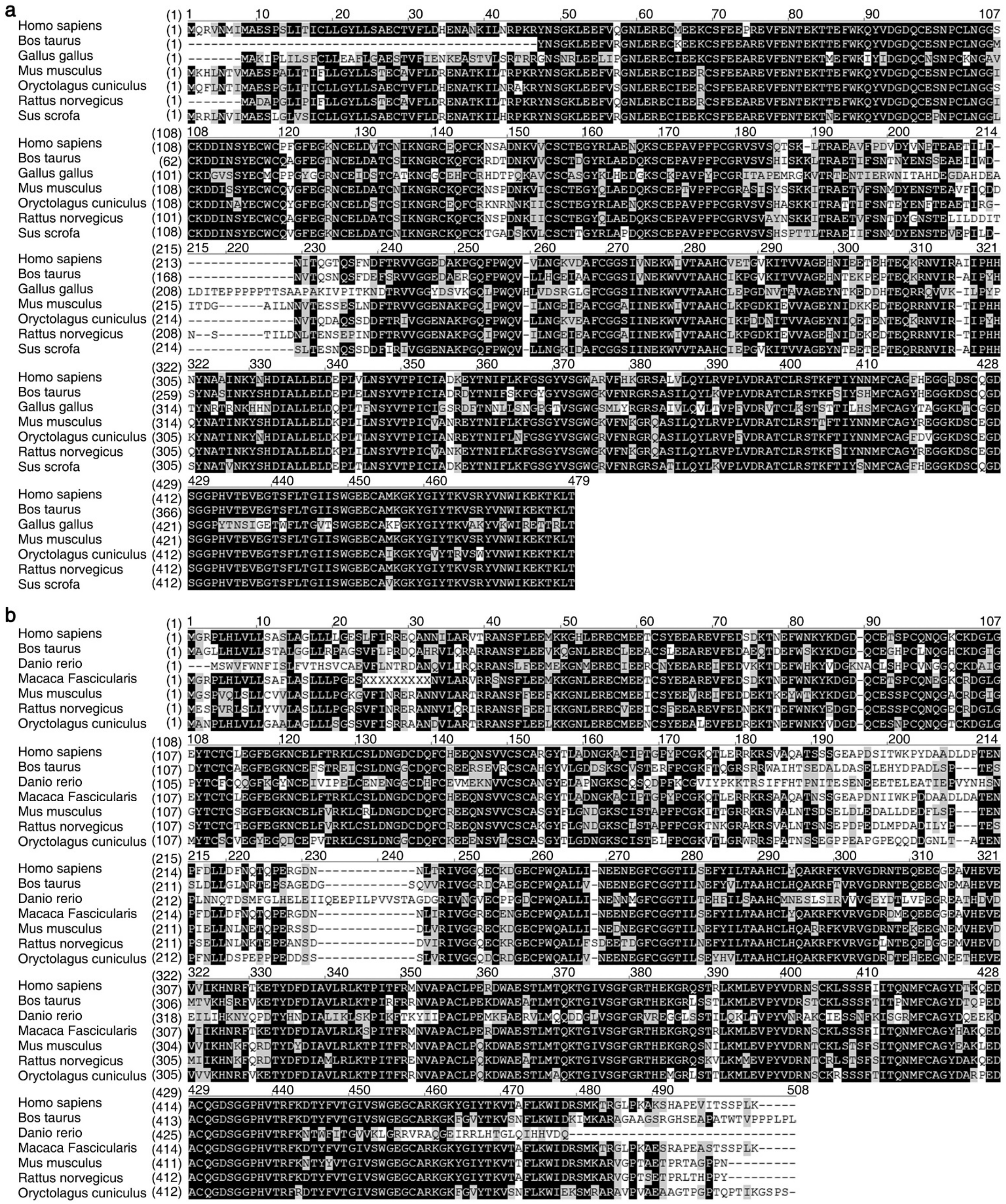

Fig. S11 Conversation of FIX (**a**) and FX (**b**) in different vertebrates. Sequence alignment was carried out using Vector Advance 11 (Invitrogen).
