# Supplementary material for: Coagulation factors VII, IX and X are effective antibacterial proteins against drug-resistant Gram-negative bacteria
Source: Cell Res. 2019 Aug 9;29(9):711–24. doi: 10.1038/s41422-019-0202-3 (PMC6796875; doi:10.1038/s41422-019-0202-3)
Supplement: Supplementary file 12 — Supplementary information, Table S1 [file 41422_2019_202_MOESM12_ESM.pdf]

Supplementary information, Table S1

Table S1 Antibacterial activities of lFVII, colistin and vancomycin towards the *A. baumannii* Ab3 (LPS+) and LPS-deficient *A. baumannii* Ab3 (LPS-). The MBC value is defined as the minimum drug concentration that causes quantitative bacterial cell death. All data are expressed as mean values ± SD (n=4).

Bacteria type	Code/name	lFVII (μM)		colistin (μM)		Vancomycin (μM)	
		LPS+	LPS-	LPS+	LPS-	LPS+	LPS-
A. baumannii	Ab3 (XDR)	0.37±0.10	3.57±0.94	0.30±0.10	10.46±5.00	20.39±10.14	0.30±0.12
