# Supplementary material for: Coagulation factors VII, IX and X are effective antibacterial proteins against drug-resistant Gram-negative bacteria
Source: Cell Res. 2019 Aug 9;29(9):711–24. doi: 10.1038/s41422-019-0202-3 (PMC6796875; doi:10.1038/s41422-019-0202-3)
Supplement: Supplementary file 13 — Supplementary information, Table S2 [file 41422_2019_202_MOESM13_ESM.pdf]

Supplementary information, Table S2

Table S2 Bacterial strains, cell lines and plasmid used in this study.

Bacterial strains		
Escherichia coli BL21(DE3)	Invitrogen	Cat# C600003
Escherichia coli DH5α	Invitrogen	Cat# 18288019
Acinetobacter baumannii ATCC 19606	ATCC	ATCC 19606
Acinetobacter baumannii Ab3	Chang et al., 2015	N/A
Acinetobacter baumannii Ab5	Chang et al., 2015	N/A
Acinetobacter baumannii Ab16	Chang et al., 2015	N/A
Acinetobacter baumannii Ab18	Chang et al., 2015	N/A
Acinetobacter baumannii Ab30	Chang et al., 2015	N/A
Acinetobacter baumannii Ab35	Chang et al., 2015	N/A
Acinetobacter baumannii Ab42	Chang et al., 2015	N/A
Acinetobacter baumannii Ab46	Chang et al., 2015	N/A
Acinetobacter baumannii Ab48	Chang et al., 2015	N/A
Klebsiella pneumonia ATCC 4352	ATCC	ATCC 4352
Enterobacter cloacae Y1	This study	N/A
Pseudomonas aeruginosa ATCC 27853	ATCC	ATCC 27853
Pseudomonas aeruginosa L93	This study	N/A
Pseudomonas aeruginosa PA3	Chang et al., 2015	N/A
Pseudomonas aeruginosa PA4	Chang et al., 2015	N/A
Cell lines		
CHO-DG44	Invitrogen	Cat# A1100001
HepG2	ATCC	ATCC HB-8065
Plasmids		
pcDNA3.1(+)	Invitrogen	N/A
pET19b	Novagen	N/A
pcDNA3.1-His-hFVII	This study	N/A
pcDNA3.1-His-FVII	This study	N/A
pcDNA3.1-His-FIX	This study	N/A
pcDNA3.1-His-FX	This study	N/A
pET19-lFVII	This study	N/A
pET19-lFIX	This study	N/A
pET19-lFX	This study	N/A
pET19-mCherry-rTEV-FVII-Gla	This study	N/A
pET19-mCherry-rTEV-FVII-EGF1	This study	N/A
pET19-mCherry-rTEV-FVII-EGF2	This study	N/A
pET19-mCherry-rTEV-FVII-EGF1mut(D1G)	This study	N/A
pET19-mCherry-rTEV-FVII-EGF1mut(G2A)	This study	N/A
pET19-mCherry-rTEV-FVII-EGF1mut(D3G)	This study	N/A
pET19-mCherry-rTEV-FVII-EGF1mut(Q4G)	This study	N/A
pET19-mCherry-rTEV-FVII-EGF1mut(C5G)	This study	N/A
pET19-mCherry-rTEV-FVII-EGF1mut(A6G)	This study	N/A
pET19-mCherry-rTEV-FVII-EGF1mut(S7G)	This study	N/A
pET19-mCherry-rTEV-FVII-EGF1mut(S8G)	This study	N/A
pET19-mCherry-rTEV-FVII-EGF1mut(P9G)	This study	N/A

pET19-mCherry-rTEV-FVII-EGF1mut(C10G)	This study	N/A
pET19-mCherry-rTEV-FVII-EGF1mut(Q11G)	This study	N/A
pET19-mCherry-rTEV-FVII-EGF1mut(N12G)	This study	N/A
pET19-mCherry-rTEV-FVII-EGF1mut(G13A)	This study	N/A
pET19-mCherry-rTEV-FVII-EGF1mut(G14A)	This study	N/A
pET19-mCherry-rTEV-FVII-EGF1mut(S15G)	This study	N/A
pET19-mCherry-rTEV-FVII-EGF1mut(C16G)	This study	N/A
pET19-mCherry-rTEV-FVII-EGF1mut(K17G)	This study	N/A
pET19-mCherry-rTEV-FVII-EGF1mut(D18G)	This study	N/A
pET19-mCherry-rTEV-FVII-EGF1mut(Q19G)	This study	N/A
pET19-mCherry-rTEV-FVII-EGF1mut(L20G)	This study	N/A
pET19-mCherry-rTEV-FVII-EGF1mut(Q21G)	This study	N/A
pET19-mCherry-rTEV-FVII-EGF1mut(S22G)	This study	N/A
pET19-mCherry-rTEV-FVII-EGF1mut(Y23G)	This study	N/A
pET19-mCherry-rTEV-FVII-EGF1mut(I24G)	This study	N/A
pET19-mCherry-rTEV-FVII-EGF1mut(C25G)	This study	N/A
pET19-mCherry-rTEV-FVII-EGF1mut(F26G)	This study	N/A
pET19-mCherry-rTEV-FVII-EGF1mut(C27G)	This study	N/A
pET19-mCherry-rTEV-FVII-EGF1mut(L28G)	This study	N/A
pET19-mCherry-rTEV-FVII-EGF1mut(P29G)	This study	N/A
pET19-mCherry-rTEV-FVII-EGF1mut(A30G)	This study	N/A
pET19-mCherry-rTEV-FVII-EGF1mut(F31G)	This study	N/A
pET19-mCherry-rTEV-FVII-EGF1mut(E32G)	This study	N/A
pET19-mCherry-rTEV-FVII-EGF1mut(G33A)	This study	N/A
pET19-mCherry-rTEV-FVII-EGF1mut(R34G)	This study	N/A
pET19-mCherry-rTEV-FVII-EGF1mut(N35G)	This study	N/A
pET19-mCherry-rTEV-FVII-EGF1mut(C36G)	This study	N/A
pET19-mCherry-rTEV-FVII-EGF1mut(E37G)	This study	N/A
