# Supplementary material for: Coagulation factors VII, IX and X are effective antibacterial proteins against drug-resistant Gram-negative bacteria
Source: Cell Res. 2019 Aug 9;29(9):711–24. doi: 10.1038/s41422-019-0202-3 (PMC6796875; doi:10.1038/s41422-019-0202-3)
Supplement: Supplementary file 14 — Supplementary information, Table S3 [file 41422_2019_202_MOESM14_ESM.pdf]

Supplementary information, Table S3

Table S3 Sequences of primers and oligos used in this study.

Name (s: sense, as: antisense)	Sequence (5′ to 3′)	Restriction Site
**Primers for FVII, FIX and FX cloning**		
FVII-s	taagctagctgcagagatttcatcatggtctcccaggccct	*Nhe* I
FVII-as	tgcggatccttagtggtggtggtggtggggaaatggggctcgcaggaggact	*Bam*H I
FIX-s	gagaagctttgcagagatttcatcatggagtttgggctgagctggctttttcttgttgctgcattaagaggtgtccagtcctata<br>attcaggtaaattggaa	*Hin*d Ⅲ
FIX-as	gccggatccttagtgatgatgatgatgatgacgggtgagcttagaagtttg	*Bam*H I
FX-s	gagaagctttgcagagatttcatcatggagtttgggctgagctggctttttcttgttgctgcattaagaggtgtccagtccgcc<br>aattcctttcttgaagag	*Hin*d Ⅲ
FX-as	gccggatccttagtgatgatgatgatgatggcgttccagggtctgtttccc	*Bam*H I
**Primers for cloning of lFVII, lFIX, lFX and hFVII**		
lFVII-s	taaccatgggccatcatcatcatcatcacgccaacgcgttcctggagga	*Nco* I
lFVII-as	tatctcgagttatcggccttggggtttgctggcatt	*Xho* I
lFIX-s	ataccatgggccatcatcatcatcatcattataattcaggtaaattggaag	*Nco* I
lFIX-as	attctcgagttaacgggtgagcttagaagtttgt	*Xho* I
lFX-s	ataccatgggccatcatcatcatcatcatgccaattcctttcttgaagag	*Nco* I
lFX-as	attctcgagttagcgttccagggtctgtttcc	*Xho* I
hFVII-s	taagctagcgccaccatgattgtgggggcaag	*Nhe* I
hFVII-as	tatggatccttagtggtggtggtggtggggaaatggggct	*Bam*H I
**Primers for cloning of FVII-Gal, FVII-EGF1 and FVII-EGF2**		
mCherry-s	taacatatggtgagcaagggcgaggaggata	*Nde* I
mCherry-as	cctggaagtacaggttttccttgtacagctcgtc	/
FVII-Gal-s	gaaaacctgtacttccagggtcaattcgaagccaacgcgttcctg	/
FVII-Gal-as	tatggatccttaactgtaagaaatccagaacagc	*Bam*H I
FVII-EGF1-s	gaaaacctgtacttccagggtcaattcgaagatggggaccagtgtgcctc	/
FVII-EGF1-as	tatggatccttactcacagttccggccctcgaa	*Bam*H I
FVII-EGF2-s	gaaaacctgtacttccagggtcaattcgaaacgcacaaggatgaccagctga	/
FVII-EGF2-as	tatctcgagttatcggccttggggtttgctggcat	*Xho* I
**Oligos for cloning of the FVII-EGF1 mutants**		
FVII-EGF1mut(D1G)-s	ttcgaaggtggggaccagtgtgcctcaagtccatgccagaatgggggctcctgcaaggaccagctccagtcctatatct<br>gcttctgcctccctgccttcgagggccggaactgtgagtaactcgag	*Bsp*119 I/*Xho* I
FVII-EGF1mut(D1G)-as	ctcgagttactcacagttccggccctcgaaggcagggaggcagaagcagatataggactggagctggtccttgcaggag<br>cccccattctggcatggacttgaggcacactggtccccaccttcgaa	
FVII-EGF1mut(G2A)-s	ttcgaagatgcagaccagtgtgcctcaagtccatgccagaatgggggctcctgcaaggaccagctccagtcctatatctg<br>cttctgcctccctgccttcgagggccggaactgtgagtaactcgag	*Bsp*119 I/*Xho* I
FVII-EGF1mut(G2A)-as	ctcgagttactcacagttccggccctcgaaggcagggaggcagaagcagatataggactggagctggtccttgcaggag<br>cccccattctggcatggacttgaggcacactggtctgcatcttcgaa	
FVII-EGF1mut(D3G)-s	ttcgaagatgggggtcagtgtgcctcaagtccatgccagaatgggggctcctgcaaggaccagctccagtcctatatctg<br>cttctgcctccctgccttcgagggccggaactgtgagtaactcgag	*Bsp*119 I/*Xho* I
FVII-EGF1mut(D3G)-as	ctcgagttactcacagttccggccctcgaaggcagggaggcagaagcagatataggactggagctggtccttgcaggag<br>cccccattctggcatggacttgaggcacactgacccccatcttcgaa	
FVII-EGF1mut(Q4G)-s	ttcgaagatggggacggttgtgcctcaagtccatgccagaatgggggctcctgcaaggaccagctccagtcctatatctg<br>cttctgcctccctgccttcgagggccggaactgtgagtaactcgag	*Bsp*119 I/*Xho* I
FVII-EGF1mut(Q4G)-as	ctcgagttactcacagttccggccctcgaaggcagggaggcagaagcagatataggactggagctggtccttgcaggag	

	cccccattctggcatggacttgaggcacaaccgtccccatcttcgaa	
FVII-EGF1mut(C5G)-s	ttcgaagatggggaccagggtgcctcaagtccatgccagaatgggggctcctgcaaggaccagctccagtcctatatctgcttctgcctccctgccttcgagggccggaactgtgagtaactcgag	*Bsp*119 I/*Xho* I
FVII-EGF1mut(C5G)-as	ctcgagttactcacagttccggccctcgaaggcagggaggcagaagcagatataggactggagctggtccttgcaggagcccccattctggcatggacttgaggcaccctggtccccatcttcgaa	
FVII-EGF1mut(A6G)-s	ttcgaagatggggaccagtgtggttcaagtccatgccagaatgggggctcctgcaaggaccagctccagtcctatatctgcttctgcctccctgccttcgagggccggaactgtgagtaactcgag	*Bsp*119 I/*Xho* I
FVII-EGF1mut(A6G)-as	ctcgagttactcacagttccggccctcgaaggcagggaggcagaagcagatataggactggagctggtccttgcaggagcccccattctggcatggacttgaaccacactggtccccatcttcgaa	
FVII-EGF1mut(S7G)-s	ttcgaagatggggaccagtgtgccggtagtccatgccagaatgggggctcctgcaaggaccagctccagtcctatatctgcttctgcctccctgccttcgagggccggaactgtgagtaactcgag	*Bsp*119 I/*Xho* I
FVII-EGF1mut(S7G)-as	ctcgagttactcacagttccggccctcgaaggcagggaggcagaagcagatataggactggagctggtccttgcaggagcccccattctggcatggactaccggcacactggtccccatcttcgaa	
FVII-EGF1mut(S8G)-s	ttcgaagatggggaccagtgtgcctcaggtccatgccagaatgggggctcctgcaaggaccagctccagtcctatatctgcttctgcctccctgccttcgagggccggaactgtgagtaactcgag	*Bsp*119 I/*Xho* I
FVII-EGF1mut(S8G)-as	ctcgagttactcacagttccggccctcgaaggcagggaggcagaagcagatataggactggagctggtccttgcaggagcccccattctggcatggacctgaggcacactggtccccatcttcgaa	
FVII-EGF1mut(P9G)-s	ttcgaagatggggaccagtgtgcctcaagtggttgccagaatgggggctcctgcaaggaccagctccagtcctatatctgcttctgcctccctgccttcgagggccggaactgtgagtaactcgag	*Bsp*119 I/*Xho* I
FVII-EGF1mut(P9G)-as	ctcgagttactcacagttccggccctcgaaggcagggaggcagaagcagatataggactggagctggtccttgcaggagcccccattctggcaaccacttgaggcacactggtccccatcttcgaa	
FVII-EGF1mut(C10G)-s	ttcgaagatggggaccagtgtgcctcaagtccaggtcagaatgggggctcctgcaaggaccagctccagtcctatatctgcttctgcctccctgccttcgagggccggaactgtgagtaactcgag	*Bsp*119 I/*Xho* I
FVII-EGF1mut(C10G)-as	ctcgagttactcacagttccggccctcgaaggcagggaggcagaagcagatataggactggagctggtccttgcaggagcccccattctgacctggacttgaggcacactggtccccatcttcgaa	
FVII-EGF1mut(Q11G)-s	ttcgaagatggggaccagtgtgcctcaagtccatgcggtaatgggggctcctgcaaggaccagctccagtcctatatctgcttctgcctccctgccttcgagggccggaactgtgagtaactcgag	*Bsp*119 I/*Xho* I
FVII-EGF1mut(Q11G)-as	ctcgagttactcacagttccggccctcgaaggcagggaggcagaagcagatataggactggagctggtccttgcaggagcccccattaccgcatggacttgaggcacactggtccccatcttcgaa	
FVII-EGF1mut(N12G)-s	ttcgaagatggggaccagtgtgcctcaagtccatgccagggtgggggctcctgcaaggaccagctccagtcctatatctgcttctgcctccctgccttcgagggccggaactgtgagtaactcgag	*Bsp*119 I/*Xho* I
FVII-EGF1mut(N12G)-as	ctcgagttactcacagttccggccctcgaaggcagggaggcagaagcagatataggactggagctggtccttgcaggagcccccaccctggcatggacttgaggcacactggtccccatcttcgaa	
FVII-EGF1mut(G13A)-s	ttcgaagatggggaccagtgtgcctcaagtccatgccagaatgcaggctcctgcaaggaccagctccagtcctatatctgcttctgcctccctgccttcgagggccggaactgtgagtaactcgag	*Bsp*119 I/*Xho* I
FVII-EGF1mut(G13A)-as	ctcgagttactcacagttccggccctcgaaggcagggaggcagaagcagatataggactggagctggtccttgcaggagcctgcattctggcatggacttgaggcacactggtccccatcttcgaa	
FVII-EGF1mut(G14A)-s	ttcgaagatggggaccagtgtgcctcaagtccatgccagaatggggcatcctgcaaggaccagctccagtcctatatctgcttctgcctccctgccttcgagggccggaactgtgagtaactcgag	*Bsp*119 I/*Xho* I
FVII-EGF1mut(G14A)-as	ctcgagttactcacagttccggccctcgaaggcagggaggcagaagcagatataggactggagctggtccttgcaggatgccccattctggcatggacttgaggcacactggtccccatcttcgaa	
FVII-EGF1mut(S15G)-s	ttcgaagatggggaccagtgtgcctcaagtccatgccagaatgggggcggttgcaaggaccagctccagtcctatatctgcttctgcctccctgccttcgagggccggaactgtgagtaactcgag	*Bsp*119 I/*Xho* I
FVII-EGF1mut(S15G)-as	ctcgagttactcacagttccggccctcgaaggcagggaggcagaagcagatataggactggagctggtccttgcaaccgcccccattctggcatggacttgaggcacactggtccccatcttcgaa	
FVII-EGF1mut(C16G)-s	ttcgaagatggggaccagtgtgcctcaagtccatgccagaatgggggctccggtaaggaccagctccagtcctatatctgcttctgcctccctgccttcgagggccggaactgtgagtaactcgag	*Bsp*119 I/*Xho* I
FVII-EGF1mut(C16G)-as	ctcgagttactcacagttccggccctcgaaggcagggaggcagaagcagatataggactggagctggtccttaccggag	

	cccccattctggcatggacttgaggcacactggtccccatcttcgaa	
FVII-EGF1mut(K17G)-s	ttcgaagatggggaccagtgtgcctcaagtccatgccagaatgggggctcctgcggtgaccagctccagtcctatatctgcttctgcctccctgccttcgagggccggaactgtgagtaactcgag	*Bsp*119 I/*Xho* I
FVII-EGF1mut(K17G)-as	ctcgagttactcacagttccggccctcgaaggcagggaggcagaagcagatataggactggagctggtcaccgcaggagcccccattctggcatggacttgaggcacactggtccccatcttcgaa	
FVII-EGF1mut(D18G)-s	ttcgaagatggggaccagtgtgcctcaagtccatgccagaatgggggctcctgcaagggtcagctccagtcctatatctgcttctgcctccctgccttcgagggccggaactgtgagtaactcgag	*Bsp*119 I/*Xho* I
FVII-EGF1mut(D18G)-as	ctcgagttactcacagttccggccctcgaaggcagggaggcagaagcagatataggactggagctgacccttgcaggagcccccattctggcatggacttgaggcacactggtccccatcttcgaa	
FVII-EGF1mut(Q19G)-s	ttcgaagatggggaccagtgtgcctcaagtccatgccagaatgggggctcctgcaaggacggtctccagtcctatatctgcttctgcctccctgccttcgagggccggaactgtgagtaactcgag	*Bsp*119 I/*Xho* I
FVII-EGF1mut(Q19G)-as	ctcgagttactcacagttccggccctcgaaggcagggaggcagaagcagatataggactggagaccgtccttgcaggagcccccattctggcatggacttgaggcacactggtccccatcttcgaa	
FVII-EGF1mut(L20G)-s	ttcgaagatggggaccagtgtgcctcaagtccatgccagaatgggggctcctgcaaggaccagggtcagtcctatatctgcttctgcctccctgccttcgagggccggaactgtgagtaactcgag	*Bsp*119 I/*Xho* I
FVII-EGF1mut(L20G)-as	ctcgagttactcacagttccggccctcgaaggcagggaggcagaagcagatataggactgaccctggtccttgcaggagcccccattctggcatggacttgaggcacactggtccccatcttcgaa	
FVII-EGF1mut(Q21G)-s	ttcgaagatggggaccagtgtgcctcaagtccatgccagaatgggggctcctgcaaggaccagctcggttcctatatctgcttctgcctccctgccttcgagggccggaactgtgagtaactcgag	*Bsp*119 I/*Xho* I
FVII-EGF1mut(Q21G)-as	ctcgagttactcacagttccggccctcgaaggcagggaggcagaagcagatataggaaccgagctggtccttgcaggagcccccattctggcatggacttgaggcacactggtccccatcttcgaa	
FVII-EGF1mut(S22G)-s	ttcgaagatggggaccagtgtgcctcaagtccatgccagaatgggggctcctgcaaggaccagctccagggttatatctgcttctgcctccctgccttcgagggccggaactgtgagtaactcgag	*Bsp*119 I/*Xho* I
FVII-EGF1mut(S22G)-as	ctcgagttactcacagttccggccctcgaaggcagggaggcagaagcagatataaccctggagctggtccttgcaggagcccccattctggcatggacttgaggcacactggtccccatcttcgaa	
FVII-EGF1mut(Y23G)-s	ttcgaagatggggaccagtgtgcctcaagtccatgccagaatgggggctcctgcaaggaccagctccagtccggtatctgcttctgcctccctgccttcgagggccggaactgtgagtaactcgag	*Bsp*119 I/*Xho* I
FVII-EGF1mut(Y23G)-as	ctcgagttactcacagttccggccctcgaaggcagggaggcagaagcagataccggactggagctggtccttgcaggagcccccattctggcatggacttgaggcacactggtccccatcttcgaa	
FVII-EGF1mut(I24G)-s	ttcgaagatggggaccagtgtgcctcaagtccatgccagaatgggggctcctgcaaggaccagctccagtcctatggttgcttctgcctccctgccttcgagggccggaactgtgagtaactcgag	*Bsp*119 I/*Xho* I
FVII-EGF1mut(I24G)-as	ctcgagttactcacagttccggccctcgaaggcagggaggcagaagcaaccataggactggagctggtccttgcaggagcccccattctggcatggacttgaggcacactggtccccatcttcgaa	
FVII-EGF1mut(C25G)-s	ttcgaagatggggaccagtgtgcctcaagtccatgccagaatgggggctcctgcaaggaccagctccagtcctatatcggtttctgcctccctgccttcgagggccggaactgtgagtaactcgag	*Bsp*119 I/*Xho* I
FVII-EGF1mut(C25G)-as	ctcgagttactcacagttccggccctcgaaggcagggaggcagaaaccgatataggactggagctggtccttgcaggagcccccattctggcatggacttgaggcacactggtccccatcttcgaa	
FVII-EGF1mut(F26G)-s	ttcgaagatggggaccagtgtgcctcaagtccatgccagaatgggggctcctgcaaggaccagctccagtcctatatctgcggttgcctccctgccttcgagggccggaactgtgagtaactcgag	*Bsp*119 I/*Xho* I
FVII-EGF1mut(F26G)-as	ctcgagttactcacagttccggccctcgaaggcagggaggcaaccgcagatataggactggagctggtccttgcaggagcccccattctggcatggacttgaggcacactggtccccatcttcgaa	
FVII-EGF1mut(C27G)-s	ttcgaagatggggaccagtgtgcctcaagtccatgccagaatgggggctcctgcaaggaccagctccagtcctatatctgcttcggtctccctgccttcgagggccggaactgtgagtaactcgag	*Bsp*119 I/*Xho* I
FVII-EGF1mut(C27G)-as	ctcgagttactcacagttccggccctcgaaggcagggagaccgaagcagatataggactggagctggtccttgcaggagcccccattctggcatggacttgaggcacactggtccccatcttcgaa	
FVII-EGF1mut(L28G)-s	ttcgaagatggggaccagtgtgcctcaagtccatgccagaatgggggctcctgcaaggaccagctccagtcctatatctgcttctgcggtcctgccttcgagggccggaactgtgagtaactcgag	*Bsp*119 I/*Xho* I
FVII-EGF1mut(L28G)-as	ctcgagttactcacagttccggccctcgaaggcaggaccgcagaagcagatataggactggagctggtccttgcaggag	

	cccccattctggcatggacttgaggcacactggtccccatcttcgaa	
FVII-EGF1mut(P29G)-s	ttcgaagatggggaccagtgtgcctcaagtccatgccagaatgggggctcctgcaaggaccagctccagtcctatatctg<br>cttctgcctcggtgccttcgagggccggaactgtgagtaactcgag	*Bsp*119 I/*Xho* I
FVII-EGF1mut(P29G)-as	ctcgagttactcacagttccggccctcgaaggcaccgaggcagaagcagatataggactggagctggtccttgcaggag<br>cccccattctggcatggacttgaggcacactggtccccatcttcgaa	
FVII-EGF1mut(A30G)-s	ttcgaagatggggaccagtgtgcctcaagtccatgccagaatgggggctcctgcaaggaccagctccagtcctatatctg<br>cttctgcctccctggtttcgagggccggaactgtgagtaactcgag	*Bsp*119 I/*Xho* I
FVII-EGF1mut(A30G)-as	ctcgagttactcacagttccggccctcgaaaccagggaggcagaagcagatataggactggagctggtccttgcaggag<br>cccccattctggcatggacttgaggcacactggtccccatcttcgaa	
FVII-EGF1mut(F31G)-s	ttcgaagatggggaccagtgtgcctcaagtccatgccagaatgggggctcctgcaaggaccagctccagtcctatatctg<br>cttctgcctccctgccggtgagggccggaactgtgagtaactcgag	*Bsp*119 I/*Xho* I
FVII-EGF1mut(F31G)-as	ctcgagttactcacagttccggccctcaccggcagggaggcagaagcagatataggactggagctggtccttgcaggag<br>cccccattctggcatggacttgaggcacactggtccccatcttcgaa	
FVII-EGF1mut(E32G)-s	ttcgaagatggggaccagtgtgcctcaagtccatgccagaatgggggctcctgcaaggaccagctccagtcctatatctg<br>cttctgcctccctgccttcggtggccggaactgtgagtaactcgag	*Bsp*119 I/*Xho* I
FVII-EGF1mut(E32G)-as	ctcgagttactcacagttccggccaccgaaggcagggaggcagaagcagatataggactggagctggtccttgcagga<br>gcccccattctggcatggacttgaggcacactggtccccatcttcgaa	
FVII-EGF1mut(G33A)-s	ttcgaagatggggaccagtgtgcctcaagtccatgccagaatgggggctcctgcaaggaccagctccagtcctatatctg<br>cttctgcctccctgccttcgaggcacggaactgtgagtaactcgag	*Bsp*119 I/*Xho* I
FVII-EGF1mut(G33A)-as	ctcgagttactcacagttccgtgcctcgaaggcagggaggcagaagcagatataggactggagctggtccttgcaggag<br>cccccattctggcatggacttgaggcacactggtccccatcttcgaa	
FVII-EGF1mut(R34G)-s	ttcgaagatggggaccagtgtgcctcaagtccatgccagaatgggggctcctgcaaggaccagctccagtcctatatctg<br>cttctgcctccctgccttcgagggcggtaactgtgagtaactcgag	*Bsp*119 I/*Xho* I
FVII-EGF1mut(R34G)-as	ctcgagttactcacagttaccgccctcgaaggcagggaggcagaagcagatataggactggagctggtccttgcaggag<br>cccccattctggcatggacttgaggcacactggtccccatcttcgaa	
FVII-EGF1mut(N35G)-s	ttcgaagatggggaccagtgtgcctcaagtccatgccagaatgggggctcctgcaaggaccagctccagtcctatatctg<br>cttctgcctccctgccttcgagggccggggttgtgagtaactcgag	*Bsp*119 I/*Xho* I
FVII-EGF1mut(N35G)-as	ctcgagttactcacaaccccggccctcgaaggcagggaggcagaagcagatataggactggagctggtccttgcagga<br>gcccccattctggcatggacttgaggcacactggtccccatcttcgaa	
FVII-EGF1mut(C36G)-s	ttcgaagatggggaccagtgtgcctcaagtccatgccagaatgggggctcctgcaaggaccagctccagtcctatatctg<br>cttctgcctccctgccttcgagggccggaacggtgagtaactcgag	*Bsp*119 I/*Xho* I
FVII-EGF1mut(C36G)-as	ctcgagttactcaccgttccggccctcgaaggcagggaggcagaagcagatataggactggagctggtccttgcagga<br>gcccccattctggcatggacttgaggcacactggtccccatcttcgaa	
FVII-EGF1mut(E37G)-s	ttcgaagatggggaccagtgtgcctcaagtccatgccagaatgggggctcctgcaaggaccagctccagtcctatatctg<br>cttctgcctccctgccttcgagggccggaactgtggttaactcgag	*Bsp*119 I/*Xho* I
FVII-EGF1mut(E37G)-as	ctcgagttaaccacagttccggccctcgaaggcagggaggcagaagcagatataggactggagctggtccttgcagga<br>gcccccattctggcatggacttgaggcacactggtccccatcttcgaa	
